# Preventing tumor progression to the bone by induced tumor-suppressing MSCs

**DOI:** 10.7150/thno.58779

**Published:** 2021-03-05

**Authors:** Xun Sun, Kexin Li, Rongrong Zha, Shengzhi Liu, Yao Fan, Di Wu, Misato Hase, Uma K. Aryal, Chien-Chi Lin, Bai-Yan Li, Hiroki Yokota

**Affiliations:** 1 Department of Pharmacology, College of Pharmacy, Harbin Medical University, Harbin 150081, China.; 2 Department of Biomedical Engineering, Indiana University Purdue University Indianapolis, Indianapolis, IN 46202, USA.; 3 Graduate School of Engineering, Mie University, Mie 514, Japan.; 4 Department of Comparative Pathobiology, Purdue University, West Lafayette, IN 47907, USA.; 5 Simon Cancer Center, Indiana University School of Medicine, Indianapolis, IN 46202, USA.; 6 Indiana Center for Musculoskeletal Health, Indiana University School of Medicine, Indianapolis, IN 46202, USA.

**Keywords:** breast cancer bone metastasis, MSCs, Lrp5, Snail, Akt

## Abstract

**Background:** Advanced breast cancer metastasizes to many organs including bone, but few effective treatments are available. Here we report that induced tumor-suppressing (iTS) MSCs protected bone from metastases while un-induced MSCs did not.

**Methods:** iTS MSCs were generated by overexpressing Lrp5, β-catenin, Snail, or Akt. Their tumor-suppressing capability was tested using a mouse model of mammary tumors and bone metastasis, human breast cancer tissues and cancer cell lines.

**Results:** In a mouse model, the induced MSC-derived conditioned medium (MSC CM) reduced mammary tumors and suppressed tumor-induced osteolysis. Tumor-promoting genes such as CXCL2 and LIF, as well as PDL1, a blocker of T-cell-based immune responses were downregulated. Proteomics analysis revealed that heat shock protein 90 (Hsp90ab1), calreticulin (Calr) and peptidylprolyl isomerase B (Ppib), which are highly expressed intracellular proteins in many cancers, were enriched in MSC CM as atypical tumor suppressors. Thus, overexpressing selected genes that were otherwise tumorigenic rendered MSCs the tumor-suppressing capability through the atypical suppressors, as well as p53 and Trail. Notably, the inhibitory effect of Lrp5- and Akt-overexpressing MSC CMs, Hsp90ab1 and Calr presented selective inhibition to tumor cells than non-tumor cells. The development of bone-resorbing osteoclasts was also suppressed by MSC CMs.

**Conclusion:** Collectively, the results showed an anti-tumor effect of iTS MSCs and suggested novel therapeutic approaches to suppress the progression of tumors into the bone.

## Introduction

Advanced breast cancer metastasizes to nearby lymph nodes and organs such as the liver, lungs, brain and bone 1. Bone is one of the most common sites of metastases 2, causing pain, fracture, and hypercalcemia 3. Current treatment options for bone metastases are limited to the administration of bisphosphonates and radionuclides, local radiation, and orthopedic surgery 4, 5. Although these therapeutic strategies may reduce medical complications and improve the quality of life, existing treatments are palliative and not effective enough to cure metastasized bone.

When metastasized to bone, tumor cells interact with osteoblasts, osteoclasts and osteocytes. To understand bone-tumor interactions, much attention has been placed on the role of bone-resorbing osteoclasts and a vicious cycle which drives a sequence of molecular interactions to worsen tumor-induced bone destruction 6, 7. Less understood is the role of multipotent bone marrow-derived mesenchymal stem cells (MSCs) in cancer progression. MSCs are highly valuable in regenerative medicine as they can be differentiated into a multitude of cell types including bone-forming osteoblasts, chondrocytes, adipocytes and other therapeutically relevant cells. MSCs have been widely used for fracture healing and cartilage regeneration 8, 9. MSCs also exert paracrine effects to regulate the immune and inflammatory response which have been shown to promote tumor progression and metastasis 10, 11. Interestingly, it has been reported that MSCs could inhibit tumor growth either by regulating intracellular signaling or by secreting soluble factors such as DKK1 12, 13. These studies highlight the complicated nature of MSC-tumor interactions and further studies are necessary to understand whether MSCs can be used clinically for treating bone metastases.

To generate tumor-suppressing MSCs, we took a counterintuitive approach by overexpressing selected genes in MSCs that could otherwise lead to tumor cell growth and invasion, such as Lrp5, β-catenin, Snail and Akt 14, 15. When a similar approach was implemented in osteocytes, we observed that overexpression of Lrp5, a co-receptor of Wnt signaling, rendered the otherwise naïve osteocytes tumor-suppressive. This observation led us to test an unconventional hypothesis: the overexpression of potentially tumorigenic genes in MSCs may generate tumor-suppressing cells through the secretion of tumor-suppressing proteins. To test this hypothesis we selected several genes that are the common targets in chemotherapy for their overexpression in MSCs 16. The approach herein was thus atypical - akin to “fighting fire with fire.” Here we used genetically modified MSCs with tumor-suppressing capabilities as induced tumor-suppressing (iTS) MSCs and evaluated the possibility of iTS-MSC-based therapy.

This new approach of generating iTS-MSCs by activating oncogenic signaling within the naïve MSCs was tested by a series of *in vitro*, *ex vivo* and *in vivo* experiments. We further performed mass spectrometry to identify novel tumor suppressors secreted by the iTS-MSCs. Whole-genome proteomics analysis predicted the tumor-suppressing action of Hsp90ab1, calreticulin and peptidylprolyl isomerase B (Ppib). Hsp90ab1 is a heat shock protein required for stabilizing varying proteins 17 whereas calreticulin and Ppib facilitate protein folding in the endoplasmic reticulum. Notably, Hsp90ab1 is upregulated in many solid tumors and has recently been reported to promote epithelial-mesenchymal transition (EMT) in gastric cancer by activating Akt and β-catenin signaling 18. On the other hand, cell surface calreticulin is shown to present two opposing functions, stimulating phagocytosis to remove cancer cells as well as efferocytosis to silence immune responses 19. Finally, Ppib is associated with tumor progression and unfavorable survival 20. Collectively, these three intracellular proteins have been shown to promote tumor progression when they are expressed in the tumor cells. Contrary to the tumor-promoting effect of MSCs, this study evaluated a unique procedure and presented a counterintuitive outcome in the development of the MSC-based therapeutic strategy.

Since one of the primary purposes herein is to treat breast cancer metastases to the bone, we tested this concept by inhibiting the development of bone-resorbing osteoclasts. Besides the suppression of tumor progression and osteoclast development, the results indicated that MSC-derived conditioned medium (CM) and atypical tumor-suppressing proteins inhibited the expression of PDL1 - the target of anti-PD1 immunotherapy 21.

## Results

### Tumor-suppressing effects of MSCs in suspension culture

**Figure 1:**
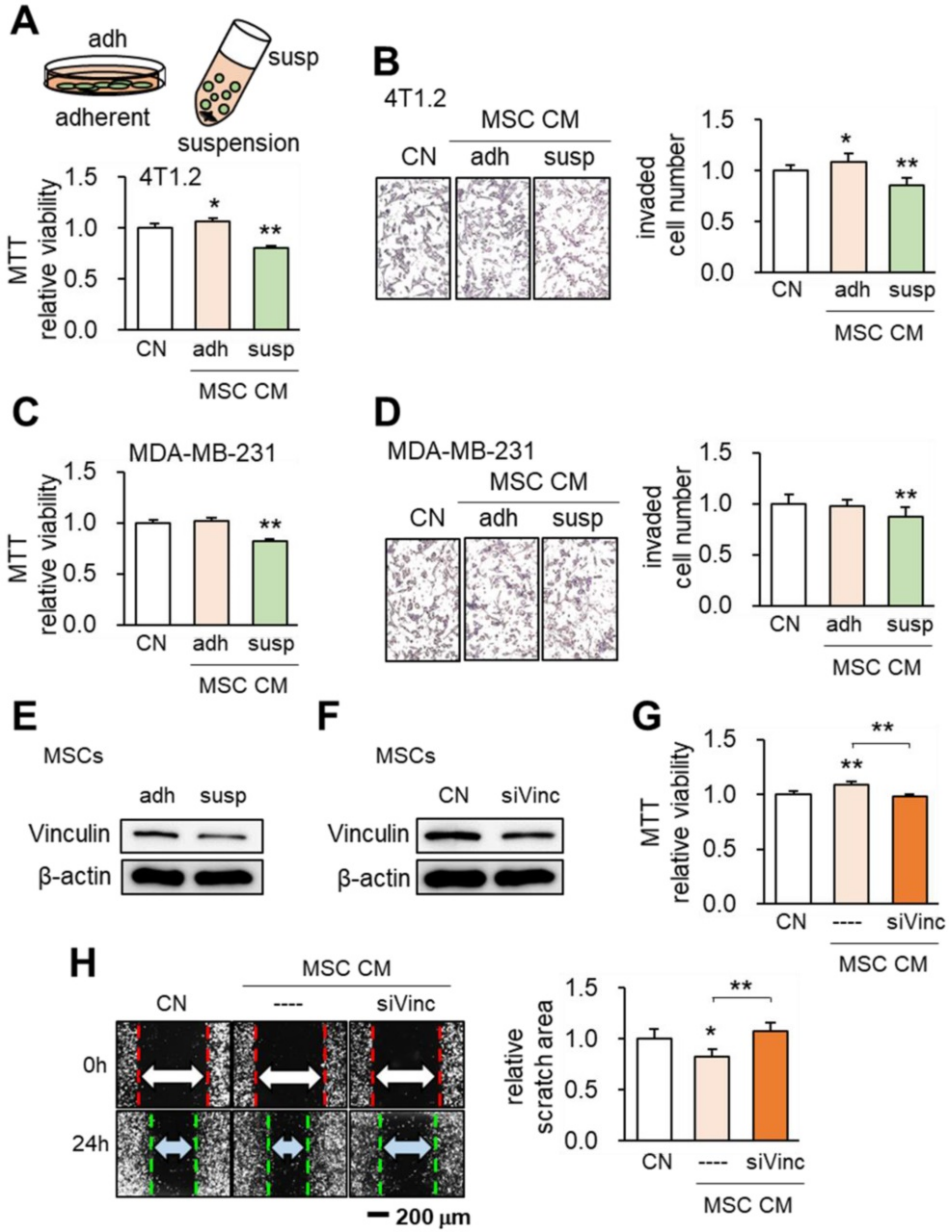
** Effects of the culture condition and vinculin on the tumor-suppressing capability of MSCs.** CN: control; adh: adherent culture; susp: suspension culture; siVinc: vinculin siRNA. The single and double asterisks indicate p < 0.05 and 0.01, respectively. **(A)** Reduction in MTT-based viability of 4T1.2 mammary tumor cells by MSC CM in suspension culture. **(B)** Decrease in the transwell-based invasion of 4T1.2 cells by MSC CM in suspension culture. **(C-D)** Inhibition of MTT-based viability and transwell-based invasion of MDA-MB-231 breast cancer cells by MSC CM in suspension culture. **(E-F)** Reduction in the level of vinculin in suspension culture and by RNA interference with vinculin siRNA. **(G-H)** Reduction in MTT-based viability and scratch-based migration of 4T1.2 cells by vinculin-silenced MSC CM.

When MSCs were cultured on the adhesive surface, the CM did not present any obvious tumor-suppressing action (Figure 1A). However when they were grown in suspension culture the CM exhibited tumor-suppressing capability by reducing MTT-based viability of 4T1.2 and EO771 mammary tumor cells (Figure 1A, Figure S1) and transwell-based invasion of 4T1.2 cells (Figure 1B), as well as MDA-MB-231 breast cancer cells (Figure 1C-D). When MSCs were cultured in suspension, Western blot analyses revealed that the expression of vinculin was reduced (Figure 1E). To evaluate the role of vinculin we silenced vinculin in MSCs using RNA interference (Figure 1F). Interestingly when vinculin-silenced MSCs were grown on the adhesive surface they gained tumor-suppressing capability as evidenced by reductions of MTT-based cell viability and scratch-based migration of 4T1.2 mammary tumor cells (Figure 1G-H). These results indicated the possibility of inducing tumor-suppressing capability in MSCs by altering the expression level of a single gene such as vinculin.

### Tumor-suppressing effects of Lrp5-overexpressing MSC CM

**Figure 2:**
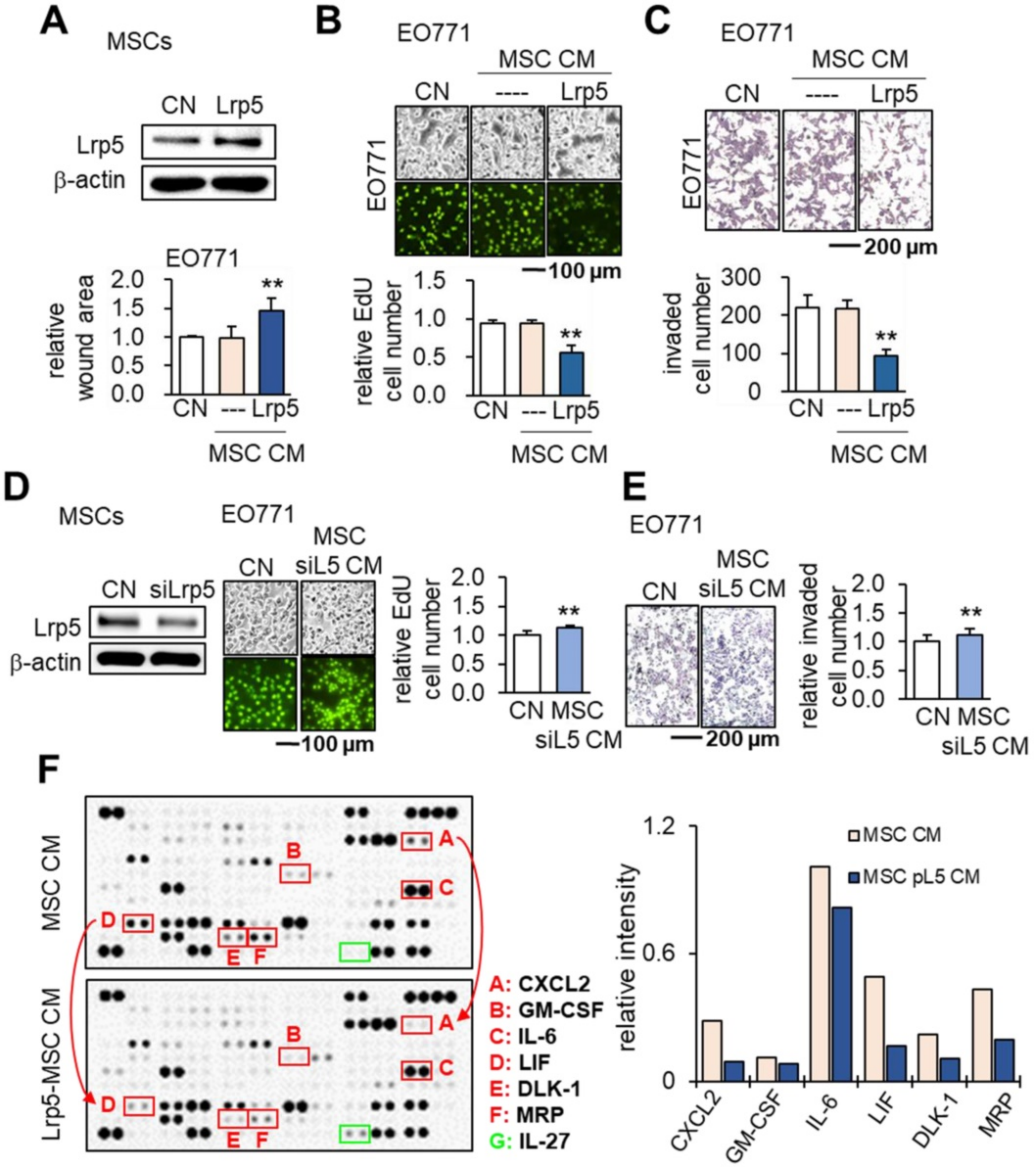
** Tumor-suppressing effects of Lrp5-overexpressing MSC CM in EO771 mammary tumor cells.** CN: control; Lrp5: Lrp5 plasmids; siL5: Lrp5 siRNA. The double asterisk indicates p < 0.01. **(A-C)** Overexpression of Lrp5 in MSCs and the inhibition of scratch-based cellular migration, EdU-based proliferation, and transwell invasion by Lrp5-overexpressing MSC CM. **(D-E)** Silencing of Lrp5 in MSCs and the increase in EdU-based proliferation and transwell invasion by Lrp5-silenced MSC CM. **(F)** Comparison of 111 cytokines and chemokines in MSC CMs with and without Lrp5 overexpression. Compared to MSC CM, Lrp5-overexpressing MSC CM elevated the levels of six proteins (CXCL2: CXC motif chemokine ligand 2; GM-CSF: granulocyte-macrophage colony-stimulating factor; IL6: interleukin 6; LIF: leukemia inhibitory factor; DLK1: delta like non-canonical Notch ligand 1; MRP: multidrug resistance protein) and reduced the level of one protein (IL27: interleukin 27).

We previously evaluated the role of Lrp5-mediated Wnt signaling in loading-driven bone formation 22. Herein, we examined whether Lrp5 would regulate the tumor-suppressing capability of MSCs. Notably Lrp5-overexpressing MSC CM reduced the scratch-based migration, EdU-based proliferation and transwell-based invasion of EO771 cells (Figure 2A-C). In response to Lrp5-overexpressing MSC CM, TRAMP prostate tumor cells also reduced EdU-based proliferation, transwell-based invasion, downregulated tumorigenic genes such as Lrp5, Runx2, MMP9 and Snail (Figure S2). By contrast, Lrp5-silenced MSC CM yielded the opposite outcome (Figure 2D-E). In cytokine and chemokine array analyses the overexpression of Lrp5 reduced the levels of several tumor-promoting factors in CM, including CXCL2, GM-CSF, IL6, LIF, DLK1 and MRP with an increase in IL27, a tumor-suppressing cytokine (Figure 2F).

### Tumor-suppressing effects of β-catenin-overexpressing MSC CMs

**Figure 3:**
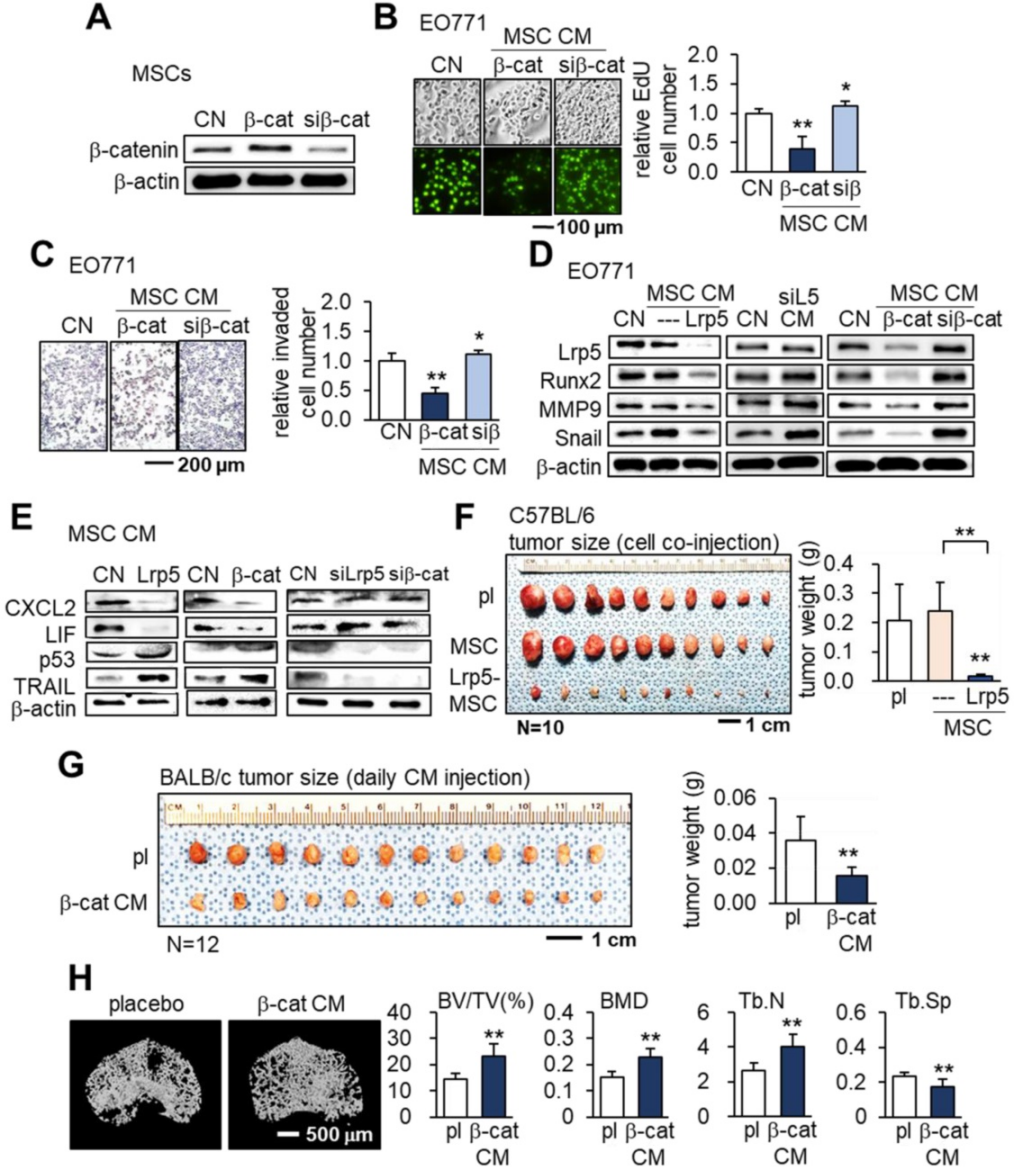
** Tumor-suppressing effects of β-catenin-overexpressing MSC CM in EO771 mammary tumor cells, C57BL/6 mice, and BALB/c mice.** CN: control; Lrp5: Lrp5 plasmids; siL5: Lrp5 siRNA; β-cat: β-catenin plasmids; siβ: β-catenin siRNA; pl: placebo. The single and double asterisks indicate p < 0.05 and 0.01, respectively. **(A-C)** Reduction in EdU-based proliferation and transwell-based invasion by β-catenin-overexpressing MSC CM, and their increase by β-catenin-silenced MSC CM. **(D)** Downregulation of Lrp5, Runx2, MMP9 and Snail in EO771 cells by Lrp5- and β-catenin-overexpressing MSC CM, and their upregulation by Lrp5- and β-catenin-silenced MSC CM. **(E)** Downregulation of CXCL2 and LIF, and upregulation of p53 and Trail in Lrp5- and β-catenin-overexpressing MSC CM. The responses are reversed by Lrp5- and β-catenin-silenced MSC CM. **(F)** Reduction in the weight of mammary tumors by the co-injection of Lrp5-overexpressing MSCs in C57BL/6 mice. **(G)** Reduction in the weight of mammary tumors by the daily intravenous administration of β-catenin-overexpressing MSC CM in BALB/c mice. **(H)** Protection of the tumor-invaded proximal tibia by the daily intravenous administration of β-catenin-overexpressing MSC CM. BV/TV: bone volume ratio; BMD: bone mineral density; Tb.N: trabecular number; Tb.Sp: trabecular separation.

We next examined the effects of β-catenin overexpression in MSCs. Similar to the results of overexpressing Lrp5, β-catenin overexpression in MSCs led to the production of CM that inhibited the proliferation and invasion of EO771 cells. On the other hand, silencing β-catenin resulted in a slight increase in EO771 cell proliferation and invasion (Figure 3A-C). Western blot analyses revealed the downregulation of oncogenic genes such as Lrp5, Runx2, MMP9 and Snail in EO771 cells cultured in CM produced by MSCs with Lrp5- or β-catenin overexpression. The expression of these oncogenic genes in EO771 cells was upregulated when the cells were cultured in CM produced by MSCs with silencing of Lrp5 or β-catenin (Figure 3D).

We then evaluated the expression of the tumor promoters CXCL2 and LIF as well as tumor suppressors p53 and Trail in EO771 cells. The overexpression of Lrp5 or β-catenin in MSCs resulted in the downregulation of CXCL2 and LIF and upregulation of p53 and Trail in their CMs, while their silencing did not (Figure 3E). In the C57BL/6 mouse model, the co-injection of Lrp5-overexpressing MSCs to the mammary fat pad significantly reduced the tumor size driven by EO771 cells (Figure 3F). We also observed the tumor-suppressing effect by the daily intravenous injection of β-catenin-overexpressing MSC CM in 4T1.2 tumor cell-inoculated BALB/c mice (Figure 3G). Furthermore, µCT imaging revealed that tumor-driven osteolysis in the proximal tibia of BALB/c mice was significantly reduced by the daily injection of β-catenin-overexpressing MSC CM (Figure 3H).

### Tumor-suppression by the overexpression of Snail in MSCs

**Figure 4:**
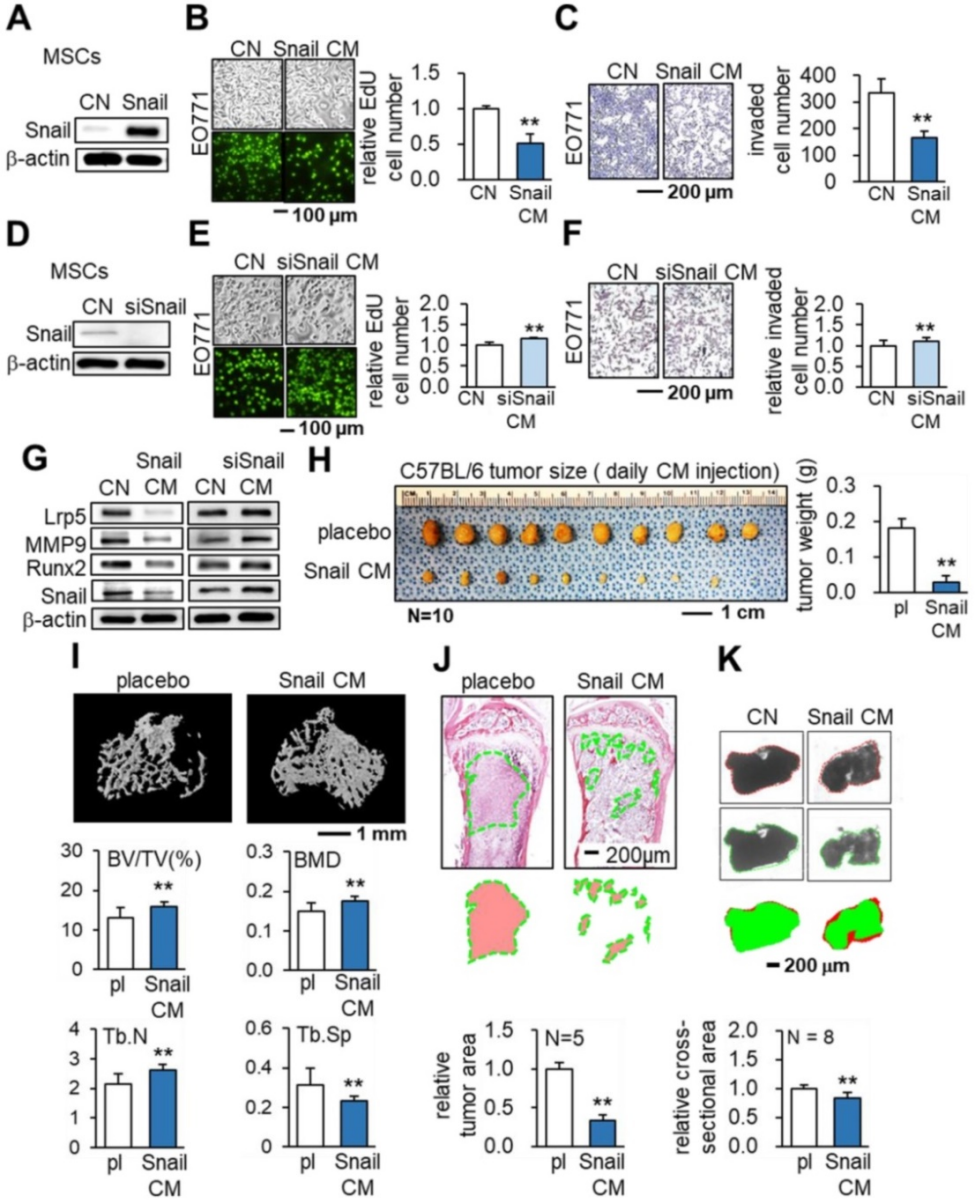
** Tumor-suppressing effects of Snail- overexpressing MSC CM in EO771 cells and C57BL/6 mice.** CN: control; Snail: Snail plasmids; siSnail: Snail siRNA. The single and double asterisks indicate p < 0.05 and 0.01, respectively. **(A-C)** Overexpression of Snail, and inhibition of EdU-based proliferation and transwell-based invasion by Snail-overexpressing MSC CM. **(D-F)** Silencing of Snail in MSCs and the increase in EdU-based proliferation and transwell invasion by Snail-silenced MSC CM. **(G)** Expression of tumorigenic genes (Lrp5, MMP9, Runx2, TGFβ and Snail) in EO771 cells in response to Snail-overexpressing and Snail-silenced MSC CMs. **(H)** Reduction in the weight of mammary tumors by the daily intravenous administration of Snail-overexpressing MSC CM. **(I)** Reduction in bone loss in the proximal tibia by the intravenous administration of Snail-overexpressing MSC CM. Of note, BV/TV: bone volume ratio; BMD: bone mineral density; Tb.N: trabecular number; Tb.Sp: trabecular separation. **(J)** Scattered distribution of EO771 tumor cells (highlighted in green) in the proximal tibia and the reduced size of tumor clusters by Snail-overexpressing MSC CM. **(K)** Shrinkage of *ex vivo* breast cancer tissue fragments by Snail-overexpressing MSC CM.

So far, we have presented evidence of the anti-tumor capability of MSC CMs by the overexpression of Lrp5 or β-catenin. We next overexpressed Snail, which was activated by Wnt signaling and is a critical mesenchymal marker in EMT. Strikingly, the overexpression of Snail also supported our hypothesis. Snail-overexpressing MSC CM reduced EdU-based proliferation (Figure 4A-B), transwell-based invasion and scratch-based migration (Figure 4C, Figure S3A), as well as the growth of EO771 tumor spheroids (Figure S3B). By contrast, Snail-silenced MSC CM reversed the responses (Figure 4D-F). The tumor-promoting genes (Lrp5, MMP9, Runx2 and Snail) in EO771 cells were decreased by treating with Snail-overexpressing MSC CMs and the effect was reversed by silencing Snail (Figure 4G). In CM produced by Snail-overexpressing MSCs, CXCL2 and LIF were significantly reduced whereas p53 and Trail were upregulated. The expression pattern was reversed by silencing Snail in MSCs (Figure S3C). In the prostate cancer cells (TRAMP), the overexpression of Snail also reduced the transwell-based invasion and downregulated the tumor-promoting genes (Figure S3D-E).

The *in vivo* result of C57BL/6 mice with EO771 cells clearly showed that daily intravenous injections of Snail-overexpressing MSC CM from the tail vein inhibited tumor progression in the mammary fat pad (Figure 4H) and the tibia (Figure 4I-J). In the proximal tibia, Snail-overexpressing MSC CM elevated the bone volume ratio (BV/TV) and bone mineral content (BMD). It also strengthened the porous trabecular bone by increasing the trabecular number (Tb.N) and decreasing the trabecular separation (Tb.Sp). Furthermore, in an *ex vivo* tissue assay with freshly isolated human breast cancer tissues, we demonstrated the shrinking of cancer tissue fragments via Snail-overexpressing MSC-CM treatment (Figure 4K).

### Tumor-suppressing capability by the overexpression of Akt in MSCs

**Figure 5:**
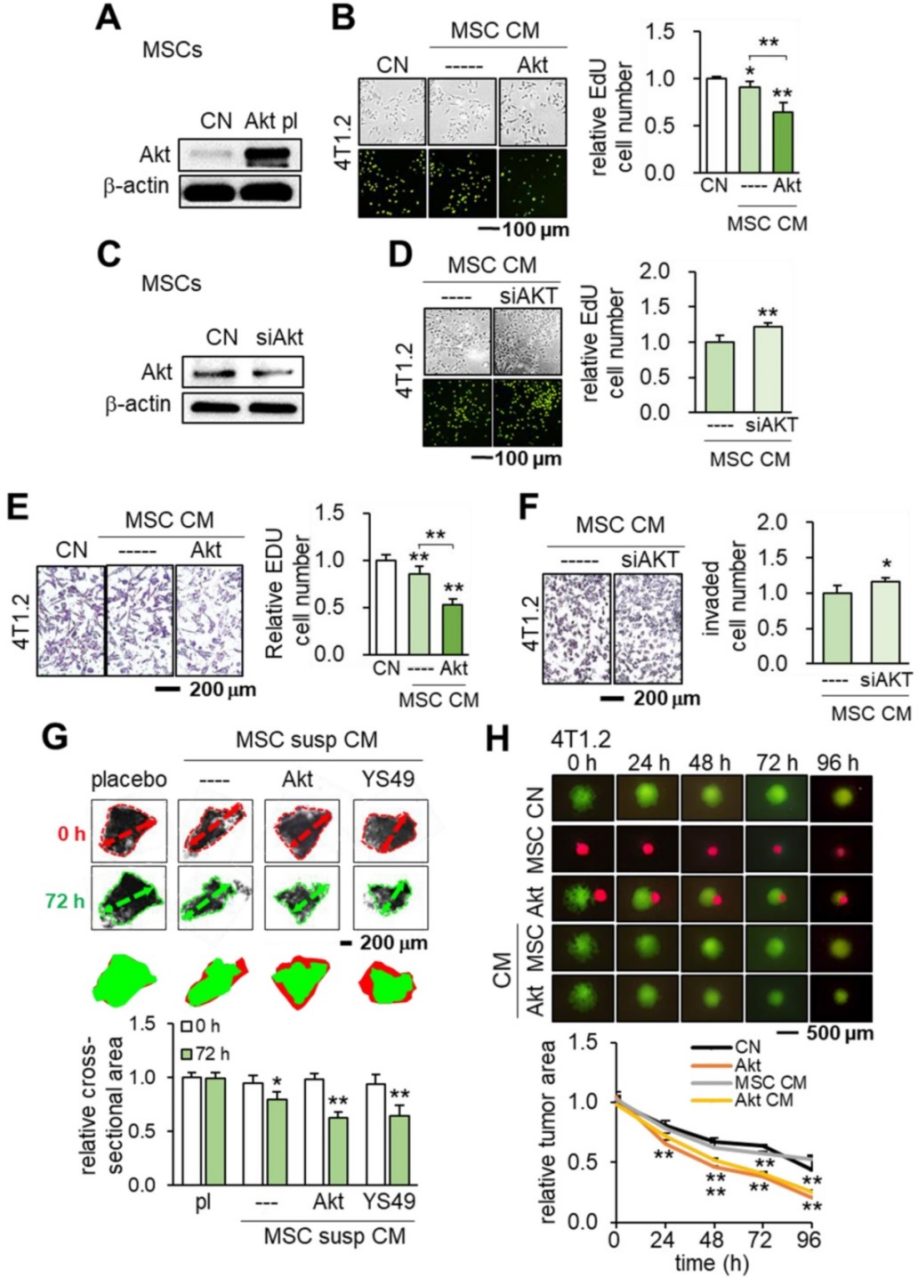
** Induction of tumor-suppressing capability by the overexpression of Akt in MSCs.** CN: control; Akt pl: Akt plasmids; siAkt: Akt siRNA. The single and double asterisks indicate p < 0.05 and 0.01, respectively. **(A-B)** Significant reduction in EdU-based proliferation of 4T1.2 cells by Akt-overexpressing MSC CM. **(C-D)** Increase in EdU-based proliferation of 4T1.2 cells by Akt-silenced MSC CM. **(E-F)** Significant decrease in transwell-based invasion of 4T1.2 cells by Akt-overexpressing MSC CM, and the opposite response by Akt-silenced MSC CM. **(G)** Shrinkage of *ex vivo* breast cancer tissues by Akt-overexpressing and YS49-treated MSC CMs. **(H)** Cell competition assay for 4T1.2 tumor spheroids in response to MSC spheroids and MSC CMs, Reduction in three-dimensional 4T1.2 tumor spheroids in response to Akt-overexpressing MSCs.

We further evaluated the effect of activating PI3K signaling by overexpressing Akt in MSCs. The results showed the same trend as seen in the overexpression of Lrp5, β-catenin or Snail. MSC CM with the overexpression of Akt reduced the EdU-based proliferation of 4T1.2 cells (Figure 5A-B) while Akt-silenced MSC CM elevated it (Figure 5C-D). Similarly, Akt-overexpressing MSC CM inhibited the transwell-based invasion of 4T1.2 cells while Akt-silenced MSC CM promoted it (Figure 5E-F).

In addition to Akt overexpression, we tested whether activating PI3K signaling via YS49, a pharmacological agent, would achieve the same anti-tumor effect. As expected, the administration of YS49 to MSCs elevated the phosphorylated Akt in MSCs (Figure S4A) while YS49-treated MSC CM reduced EdU-based proliferation and transwell-based invasion of 4T1.2 cells (Figure S4B-C). Furthermore, both Akt-overexpressing and YS49-treated MSC CMs inhibited the *ex vivo* growth of tumor fragments that were derived from freshly isolated ER-positive human breast cancer tissues (Figure 5G). Akt-overexpression and YS49-treated MSC CMs downregulated the oncogenic genes such as Lrp5, MMP9, Runx2, TGFβ and Snail in 4T1.2 cells and EO771 cells and reduced the MTT-based cell viability while Akt-silenced MSC CM elevated those genes (Figure S4D-F). The tumor-suppressing effect of Akt overexpression was observed not only in 4T1.2 cells but also in EO771 cells and MDA-MB-231 breast cancer cells (Figure S4G-H). Lastly, we conducted a spheroid competition assay and evaluated the effect of MSC-tumor interactions. The result revealed that Akt-, Lrp5-, β-catenin- and Snail-overexpressing MSCs and their CMs significantly inhibited the growth of 4T1.2 tumor spheroids (Figure 5H, Figure S5). Also, the levels of CXCL2 and LIF were lowered and those of p53 and Trail were elevated in Akt-overexpressing and YS49-treated MSC CMs (Figure S6A).

### Suppression of mammary tumors and bone degradation by Akt overexpression

**Figure 6:**
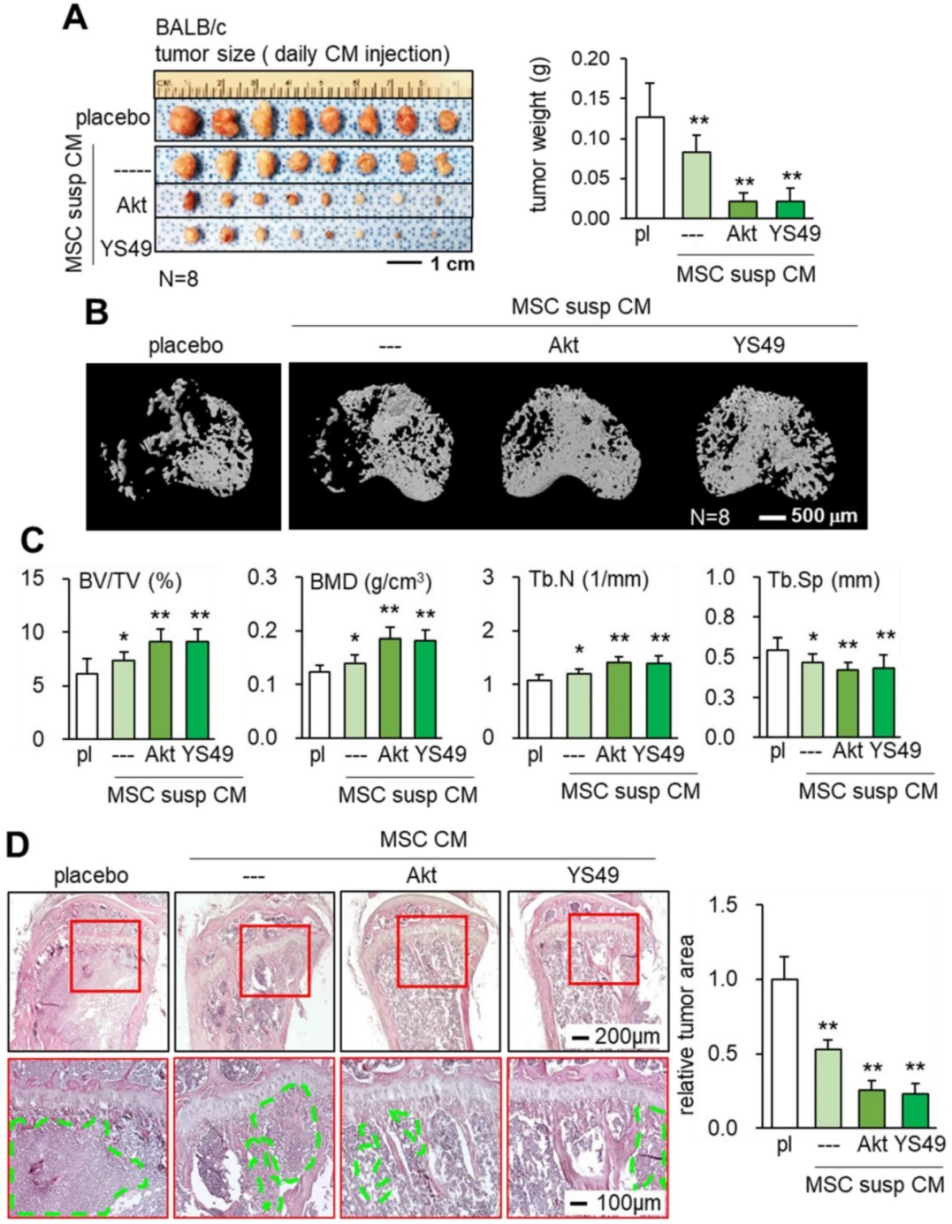
** Suppression of tumor growth in the mammary fat pad and proximal tibia in BALB/c mice by the administration of MSC CM in suspension culture,** Akt-overexpressing, and YS49-treated MSC CMs. Of note, pl: placebo (inoculation of 4T1.2 cells without treatment); susp: suspension culture. The single and double asterisks indicate p < 0.05 and 0.01, respectively. **(A)** Reduction in the weight of mammary tumors by the daily intravenous administration of MSC CM in suspension culture, and Akt-overexpressing and YS49-treated MSC CMs. **(B-C)** Protection of the tumor-invaded proximal tibia by the daily intravenous administration of MSC CMs. BV/TV: bone volume ratio; BMD: bone mineral density; Tb.N: trabecular number; Tb.Sp: trabecular separation. **(D)** Significant reduction in 4T1.2 tumor cells (highlighted in green) in the proximal tibia and the average tumor cluster size by the daily intravenous administration of MSC CM in suspension culture, and Akt-overexpressing and YS49-treated MSC CMs.

Consistent with the *in vitro* and *ex vivo* results the daily intravenous injection of Akt-overexpressing and YS49-treated MSC CMs reduced the weight of mammary tumors in 4T1.2 BALB/c mice (Figure 6A). Furthermore, bone loss in tumor-invaded tibia was suppressed with CMs produced by Akt-overexpressing and YS49 treated MSCs (Figure 6B). Furthermore, the engineered MSC CMs led to an increase in the bone volume ratio, bone mineral density and trabecular number with a concomitant decrease in trabecular separation (Figure 6C). H&E-stained histological sections revealed that the tumor-invaded area was significantly reduced by the administration of Akt-overexpressing and YS49-treated MSC CMs (Figure 6D). Collectively, these results support the protection of bone by the daily intravenous administration of MSC CMs.

### Hsp90ab1, calreticulin, and peptidylprolyl isomerase B as tumor-suppressing proteins

**Figure 7:**
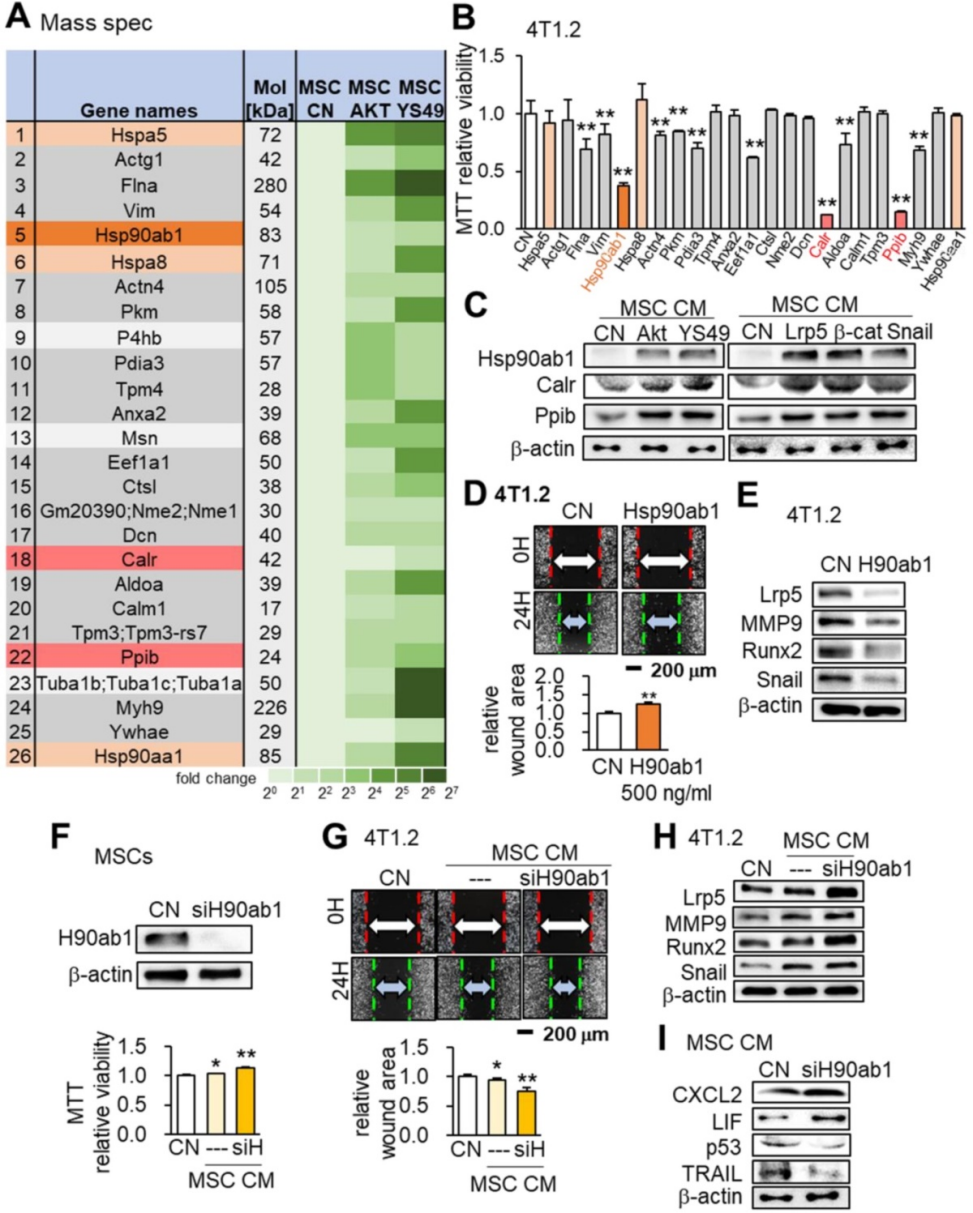
** Mass spectrometry-based proteomics analysis and the tumor-suppressing effect of Hsp90ab1.** Of note, siH or siH90ab1: Hsp90ab1 siRNA; CN: control; CM: conditioned medium. The single and double asterisks indicate p < 0.05 and 0.01, respectively. **(A)** List of the selected proteins, enriched in Akt-overexpressing and YS49-treated MSC CM. **(B)** Significant reduction in MTT-based viability of 4T1.2 cells by the incubation with 23 recombinant proteins, including Hsp90ab1, Calr and Ppib. **(C)** Upregulation of Hsp90ab1, Calr, and Ppib in MSC CMs by the overexpression of Akt, Lrp5, β-catenin, and Snail, as well as the administration of YS49 **(D)** Significant reduction in scratch-based migration of 4T1.2 cells by Hsp90ab1. **(E)** Downregulation of Lrp5, Runx2, MMP9, and Snail in 4T1.2 cells by Hsp90ab1. **(F-G)** Silencing of Hsp90ab1 in MSCs and the increase in MTT-based proliferation and scratch-based migration by Hsp90ab1-silenced MSC CM. **(H)** Increase in the levels of tumorigenic genes (Lrp5, MMP9, Runx2, TGFβ and Snail) in 4T1.2 cells in response to Hsp90ab1-silenced MSC CM. **(I)** Upregulation of CXCL2 and LIF, and downregulation of p53 and Trail in Hsp90ab1-silenced MSC CMs.

We conducted mass spectrometry-based proteomics analyses to identify the proteins in the MSC CM that were critical for the tumor-suppressing action. Focusing on the PI3K signaling pathway, we employed 4 CMs (medium control without MSCs, MSC CM control, Akt-overexpressing CM and YS49-treated CM) and identified a total of 885 proteins. There were 104 proteins identified in the Akt-overexpressing MSC CM and 75 proteins were expressed at a higher level in both Akt-overexpressing and YS49-treated CM than the control CM (Table S1). Twenty-three top candidates are listed as potential tumor suppressors, and, based on the availability of recombinant proteins, the effects of 23 proteins on the MTT-based viability of 4T1.2 tumor cells were evaluated (Figure 7A). Among the 23 proteins 11 induced statistically significant decreases in MMT-based viability (Figure 7B). Four heat shock proteins (Hspa5, Hsp90ab1, Hspa8 and Hsp90aa1) were included in the top candidate list and Hsp90ab1 was predicted to be one of the most influential tumor suppressors, as well as calreticulin (Calr) and peptidylprolyl isomerase B (Ppib). Hereafter we focused on the role of these three proteins that were upregulated in MSC CM by the overexpression of Akt, Lrp5, β-catenin and Snail and also the administration of YS49 (Figure 7C).

### Hsp90ab1 as an extracellular tumor suppressor

Hsp90ab1 reduced the scratch-based migration and downregulated tumor-promoting genes Lrp5, MMP9, Runx2 and Snail in 4T1.2 cells (Figure 7D-E). In contrast Hsp90ab1-silenced MSC CM yielded the opposite outcome (Figure 7F-H). In Hsp90ab1-silenced MSC CM the expression profile showed the elevation of CXCL2 and LIF and the reduction in p53 and Trail (Figure 7I). Furthermore, MSC CMs treated with siRNAs specific for Akt, Lrp5, β-catenin or Snail did not show elevation of Hsp90ab1, calreticulin and peptidylprolyl isomerase B (Figure S6B). Of note, the administration of heat shock protein 1 (HSF1) reduced the MTT-based viability of 4T1.2 cells whereas Hsp90aa1 recombinant protein did not alter the levels of Lrp5, MMP9, Runx2 or Snail in 4T1.2 cells (Figure S6C-D). The pharmacological inhibitor of Hsp90, Tanespimycin (17-AGG), blocked the tumor-suppressing capability of Hsp90ab1. Incubating MSCs at 42 °C for 1 h elevated the level of Hsp90ab1 and induced heat shock-driven generation of iTS MSCs (Figure S7).

### Calreticulin (Calr) and peptidylprolyl isomerase B (Ppib) as tumor suppressors

**Figure 8:**
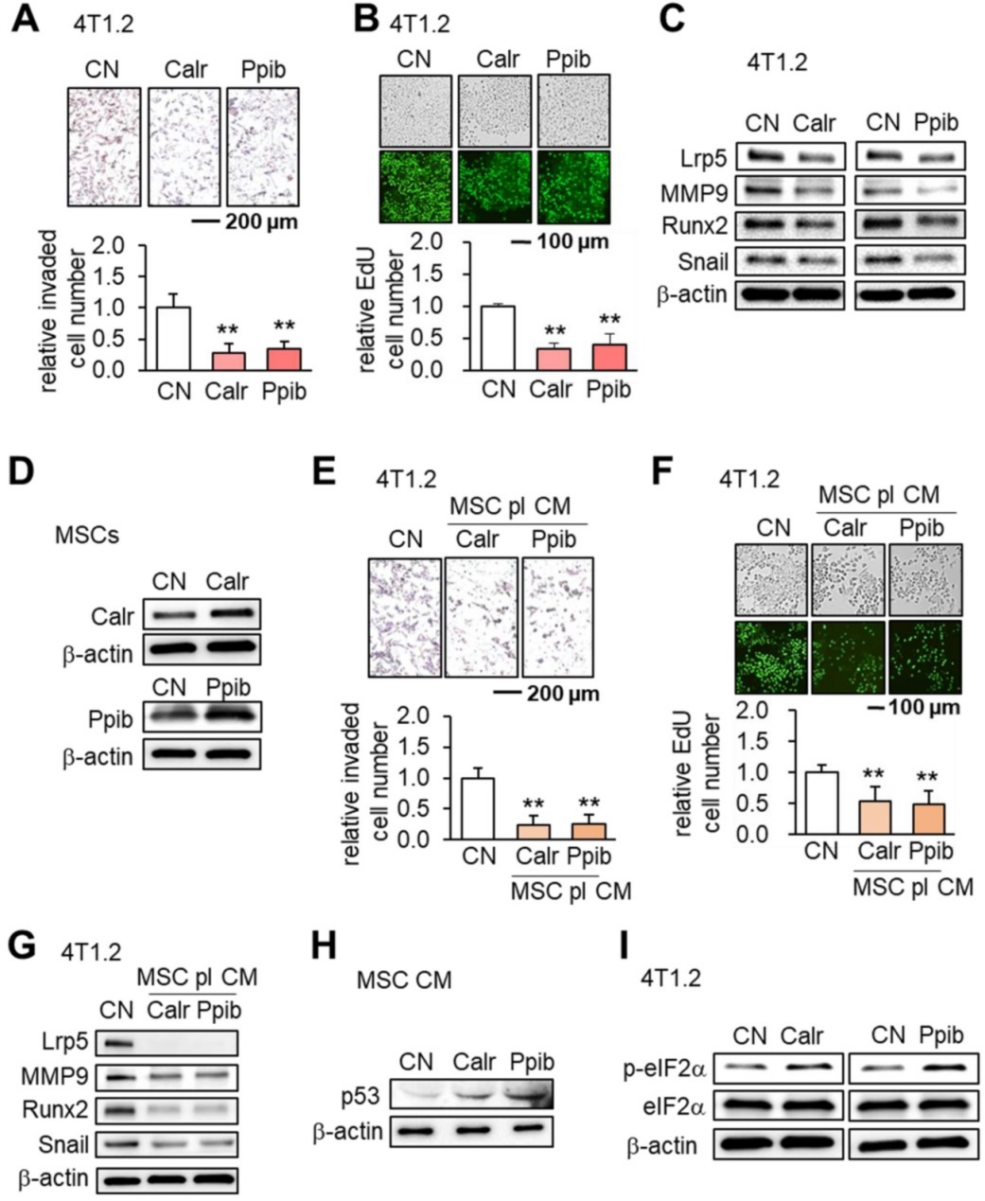
** Effects of Calr and Ppib on 4T1.2 mammary tumor cells.** The double asterisk indicates p < 0.01. CN: control; CM: conditioned medium. **(A-B)** Reduction in transwell invasion and EdU-based proliferation by recombinant Calr and Ppib proteins. **(C)** Reduction in Lrp5, MMP9, Runx2 and Snail by recombinant Calr and Ppib proteins. **(D)** Overexpression of Calr and Ppib in MSCs. **(E-F)** Reduction in Transwell invasion and EdU-based proliferation of 4T1.2 cells in response to Calr- and Ppib-overexpressing MSC CM. **(G)** Downregulation of Lrp5, MMP9, Runx2 and Snail in 4T1.2 cells in response to Calr- and Ppib-overexpressing MSC CM. **(H)** Upregulation of p53 in Calr- and Ppib-overexpressing MSC CM. **(I)** Elevation of phosphorylated eIF2a in 4T1.2 cells in response to Calr and Ppib recombinant proteins.

Calr and Ppib are proteins in the endoplasmic reticulum (ER) and facilitate protein folding and assembly. Similar to using Hsp90ab1, the addition of extracellular Calr and Ppib significantly inhibited transwell invasion and EdU-based proliferation of 4T1.2 cells (Figure 8A-B). The expression of Lrp5, MMP9, Runx2 and Snail in 4T1.2 cells was also suppressed by extracellular Calr and Ppib (Figure 8C). Notably, Calr- and Ppib-overexpressing MSC CMs significantly reduced transwell invasion and EdU-based proliferation of 4T1.2 cells (Figure 8D-F). Furthermore these MSC CMs downregulated Lrp5, MMP9, Runx2 and Snail (Figure 8G). There was also an elevation of p53 in Calr- and Ppib-overexpressing MSC CMs (Figure 8H). Stress to the ER elevates the phosphorylation level of eukaryotic translation initiation factor 2 alpha (eIF2α). Interestingly, the application of Calr and Ppib recombinant proteins to 4T1.2 mammary tumor cells elevated the phosphorylation level of eIF2α (Figure 8I).

### Tumor-selective inhibition by MSC CMs, Hsp90ab1 and Calr

**Figure 9:**
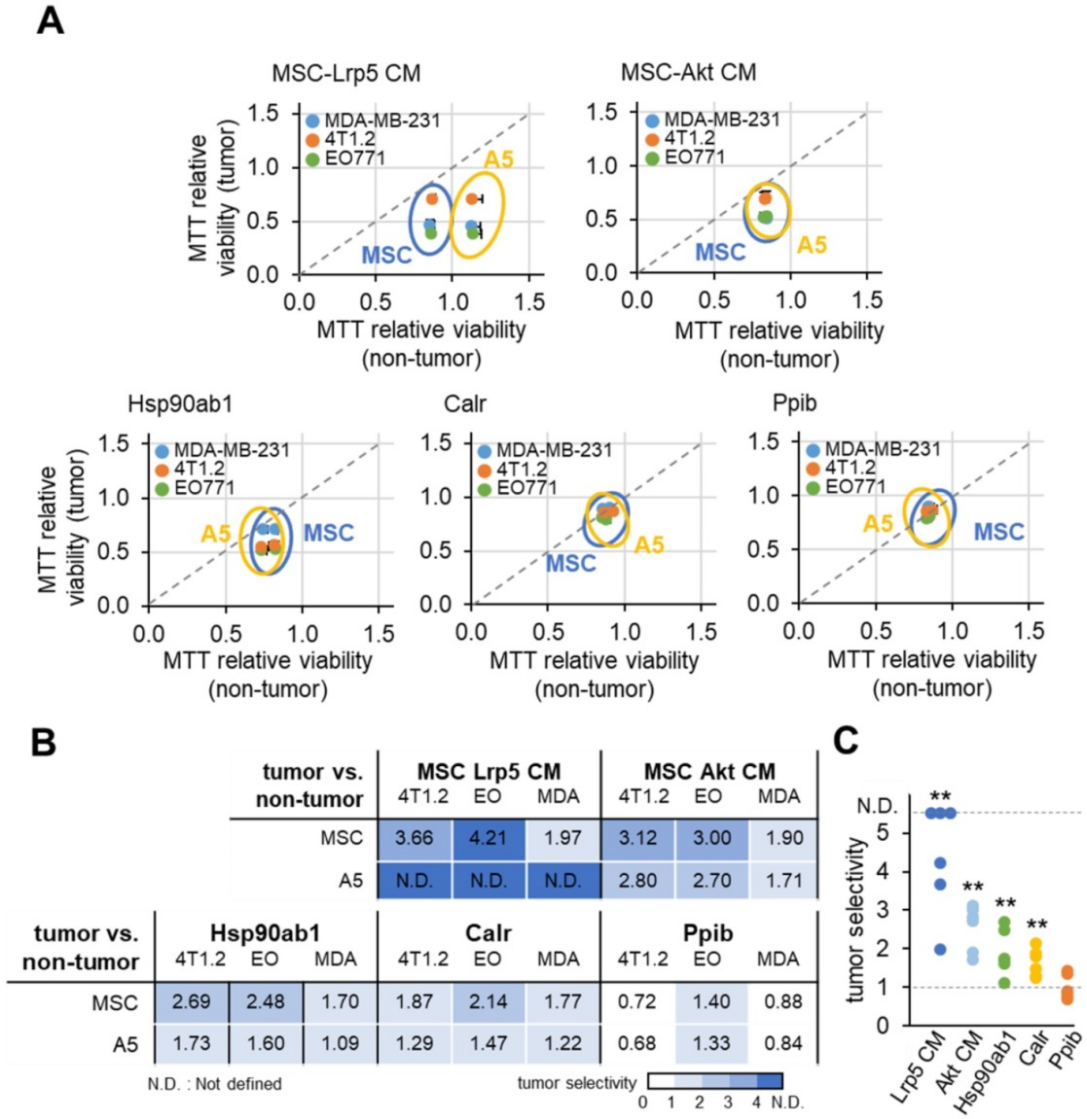
** MTT-based tumor selectivity of the inhibition of the growth of tumor cells by Lrp5 CM, Akt CM, Hsp90ab1, Calr and Ppib.** The tumor selectivity was defined as the ratio of (reduction in MTT-based viability of tumor cells) to (reduction in MTT-based viability of non-tumor cells). The double asterisk indicates p < 0.01. **(A)** Comparison of MTT-based viability of tumor cells (4T1.2, EO771, and MDA-MB-231 cells) and non-tumor cells (MSCs, and MLO-A5 osteocytes). **(B-C)** Summary of the tumor selectivity.

Ideally MSC CMs and tumor-suppressing protein candidates should only inhibit the progression of tumor cells and not normal cells. We defined the inhibitory ratio using MTT-based viability as a reduction in MTT-based viability of tumor cells compared to a reduction in MTT-based viability of non-tumor cells. A value <1 indicates that the inhibition is stronger to tumor cells than non-tumor cells. Using three tumor cells (MDA-MB-231, 4T1.2 and EO771) and two normal cells (MSCs and MLO-A5 osteocytes), we determined tumor selectivity for Lrp5 CM, Akt CM, Hsp90ab1, Calr and Ppib (Figure 9). The highest tumor selectivity was obtained with Lrp5 CM, in which the MTT-based viability of A5 osteocytes was stimulated. Akt CM also showed the tumor selectivity of 2.41 ± 0.70 and Hsp90ab1 and Calr gave a higher tumor selectivity than Ppib.

### Inhibition of osteoclast development by MSC CM

**Figure 10:**
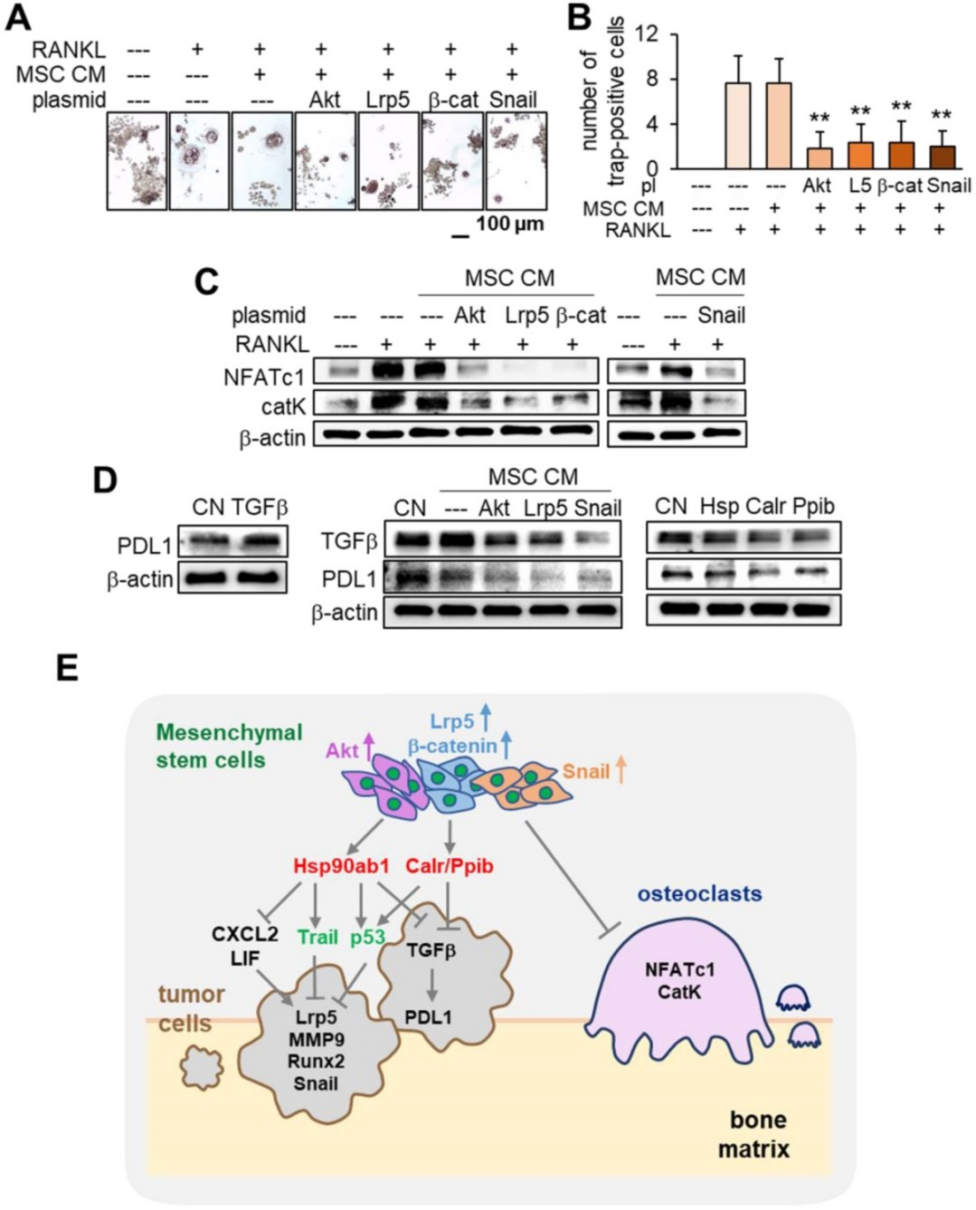
** Inhibition of osteoclast development, PDL1 expression, and the regulatory mechanism with MSC CM.** The double asterisk indicates p < 0.01. **(A-B)** Significant reduction in TRAP-positive multi-nucleated osteoclasts (> 3 nuclei) by MSC CMs with the overexpression of Akt, Lrp5, β-catenin and Snail. RAW264.7 pre-osteoclasts were stimulated their differentiation by RANKL. **(C)** Significant downregulation of NFATc1 and cathepsin K in RANKL-stimulated pre-osteoclasts by MSC CMs with the overexpression of Akt, Lrp5, β-catenin, and Snail. **(D)** Increase in PDL1 in EO771 mammary tumor cells in response to 500 ng/mL TGFβ, and decrease in TGFβ and PDL1 by MSC CM and Hsp90ab1, Calr, and Ppib. **(E)** Regulatory mechanism by MSC CM for the suppression of tumor progression and osteoclast development.

In tumor-invaded bone it is important to inhibit the development of osteoclasts that resorb bone. We examined the effect of MSC CM on the maturation and gene expression in osteoclasts. In addition to tumor suppression the results showed that MSC CMs markedly inhibited the development of RANKL-stimulated RAW264.7 pre-osteoclasts by the overexpression of Akt, Lrp5, β-catenin and Snail (Figure 10A-B). The levels of NFATc1 and cathepsin K were significantly reduced by these MSC CMs (Figure 10C). Taken together the results support the inhibitory action of MSC CM for not only tumor progression but also for the development of osteoclasts.

### Suppression of PDL1 by MSC CM

Finally we examined the effect of MSC CM on T cell-linked immune responses. PD1 is a checkpoint protein on T cells and PDL1 is an inhibitory modulator of the immune response against cancer cells. The expression of PDL1 was elevated in EO771 mammary tumor cells by the application of TGFβ. However Akt-, Lrp5- and Snail-overexpressing MSC CM, as well as Hsp90ab1, Calr and Ppib, downregulated both TGFβ and PDL1 (Figure 10D). These results indicate that MSC CM and the newly identified tumor-suppressing proteins can act as PDL1 inhibitors.

## Discussion

This study showed that MSCs were able to gain a tumor-suppressing capability by overexpressing Lrp5, β-catenin, Snail or Akt. The overexpression of these genes elevated Hsp90ab1, calreticulin, Ppib, Trail and p53 in their CMs as well as downregulated tumor-promoting cytokines such as CXCL2 and LIF as well as PDL1, a target of anti-PD1 immunotherapy. The systemic CM administration suppressed the growth of mammary tumors and tumor-driven bone loss. In addtion these CMs inhibited osteoclastogenesis by downregulating NFATc1, a master transcription factor for osteoclastogenesis, and cathepsin K, a protease for bone resorption. While MSC-derived CM has been employed to stimulate the healing of spinal cords and skin burns in regenerative medicine, to the best of our knowledge its use has not been applied to breast cancer-associated bone metastasis 23, 24. Collectively this study demonstrates an MSC CM-based therapeutic option to suppress not only tumor growth but also osteoclast development (Figure 10E).

Paradoxically the overexpression of the selected genes such as Lrp5, β-catenin, Snail and Akt are common targets of chemotherapy. However, it promoted a tumor-suppressing capability to MSC-derived CM and RNA interference using siRNAs specific to each of these genes with subsequent conversion of CMs into the tumor-promoting agents. Western blot analyses showed that MSC-derived CM consisted of the elevated level of the well-known tumor suppressor p53 as well as Trail, an apoptosis inducer to tumor cells. It was unexpected that whole-genome proteomics analyses predicted Hsp90ab1, calreticulin and Ppib as tumor suppressors since they are highly expressed in many cancer tissues. Hsp90ab1 is a chaperone protein that assists in the stabilization of a variety of proteins and its expression level is upregulated in numerous solid tumors 25. It was shown to stabilize Lrp5, to promote both EMT via activation of Akt as well as Wnt/β-catenin signaling 18. The tumor-suppressing action of extracellular HSP90ab1 in MSC-derived CM was the opposite to the action of its intracellular counterpart.

Calr and Ppib typically reside in the ER where they facilitate in protein folding and assembly. However cell surface Calr was shown to present two opposing functions: stimulation of phagocytosis to remove cancer cells and efferocytosis to silence immune responses 19. Further, Ppib was shown to be associated with tumor progression and linked to poor survival 20. Surprisingly, in this study we found that extracellularly delivered Calr and Ppib acted as potent tumor suppressors. Thus we postulate that the generation of iTS cells resembles a procedure in the evolutionarily conserved cell competition in which the fittest cells survive by eliminating lesser fit cells 26, 27. Notably Lrp5, β-catenin, Snail and Akt promoted tumor progression when they were overexpressed in tumor cells whereas MSC CMs with their overexpression acted as tumor-suppressing agents. The results indicated that multiple strategies exist to suppress tumor growth besides the traditional approach to inhibit tumorigenic pathways. As our proteomics analyses indicated, it is also conceivable that the role of the same protein can show different actions depending on whether they are located intracellulary or extracellulary.

The tumor suppressor p53 leads to apoptosis and cell cycle arrest 28. Its mutation is the most common genetic change in tumor cells 29. In the Cancer Genome Atlas database 37% of breast cancer cases show mutations in p53. We showed that overexpression of p53 and Trail were the regulators downstream of Hsp90ab1, calreticulin and Ppib. Combinatorial use of Trail with p53 was recently proposed as a novel Trail-based therapy 30, 31. We also observed in the protein array analysis that the overexpression of the four genes reduced LIF and CXCL2 while at the same time elevated IL27 in MSC CMs. LIF is a multi-functional cytokine while CXCL2 is one of the chemotactic factors known to promote inflammation and tumor growth 32, 33. IL27 has been shown to be an immune-enhancing cytokine with potent anti-tumor activity 34.

In this study we employed MSC CMs with a variety of protein having different tumor selectivities. It will be of interest to determine whether critical tumor-suppressing factors are different depending on the genes that are overexpressed. It is possible, perhaps likely, that in addition to proteins, other factors such as lipids or other extracellular vesicles are involved in tumor suppression. We also showed the incubation of CM at 42 ºC did not reduce their action for tumor suppression (data not shown) and thus the critical factors appear to be heat resistant. Of note, the molecular weight of the contributing factors in the CM are larger than 3 kDa, since filtering CM with a membrane with a cutoff at 3 kDa did not alter anti-tumor capability (data not shown). While we demonstrated that administration of recombinant Calr and Ppib inhibited tumor cells, the mixture of multiple CM-derived factors may be more advantageous owing to the potential synergistic effects of multiple factors. In addition to overexpressing Akt we also employed YS49 as a pharmacological activator of PI3K/Akt signaling. This approach may provide an easier route for converting MSCs to iTS cells.

CM derived from MSCs has been increasingly used in regenerative medicine, tissue engineering and immunotherapy 35. MSCs can differentiate into osteoblasts, chondrocytes and adipocytes. For bone regeneration MSCs are typically harvested from bone marrow owing to their strong osteogenic potential. However MSCs can also be isolated from other more assessable locations, e.g., adipose tissue, and it may be worthy of investigating their potential anti-tumor effects. We have previously shown that Lrp5 in osteocytes contributes to protecting tumor-driven bone loss 36. Hence, other MSC-derived cells, e.g., osteoblasts or osteocytes, may be used to generate iTS cells to mitigate to the potential issues by using undifferentiated MSCs.

Besides genetic modification and the administration of pharmacological agents whether other external physicochemical stimuli such as ER stress and mechanical stimulation may enhance the tumor-suppressing capability of iTS cells.

While this study employed *in vitro*, *ex vivo* and *in vivo* models with breast and prostate cancer cells, the response to MSC CMs and the degree of inhibitory effects may depend on the types of cancer cells, hormonal receptor status and p53 mutations. It is of further interest to know whether any other genes can be overexpressed to induce iTS cells and whether those options may provide unique efficacies. It is also of interest whether any other cells besides MSCs can be converted to iTS cells by the overexpression of the selected genes.

In summary, the gene overexpression approach and a systemic administration of MSC CMs effectively inhibited the growth of mammary tumors and tumor-induced osteolysis in the two mouse models. Further studies are warranted to understand the regulatory basis of the action of engineered MSCs and to evaluate the possibility of clinical translation for the treatment of tumor-invaded bone.

## Materials and Methods

### Cell culture

EO771 mouse mammary tumor cells (CH3 BioSystems, Amherst, NY, USA) 37, 4T1.2 mouse mammary tumor cells (obtained from Dr. R. Anderson at Peter MacCallum Cancer Institute, Australia) 38, and MDA-MB-231 breast cancer cells (ATCC) were cultured in DMEM. RAW264.7 pre-osteoclast cells (ATCC, Manassas, VA, USA) were grown in αMEM 39, 40. TRAMP-C2ras prostate tumor cells (ATCC) were cultured in DMEM/F-12 41, and PC-3 human prostate cancer cells (ATCC) were cultured in RPMI-1640 (Gibco, Carlsbad, CA, USA) 42. Murine MSCs derived from the bone marrow of the C57BL/6 strain (Envigo RMS, Inc., Indianapolis, IN, USA) were cultured in MesenCult culture medium (Stem Cell Technology, Cambridge, MA, USA). The culture media was supplemented with 10% fetal bovine serum and antibiotics, and cells were maintained at 37°C and 5% CO_2_. In a heat shock experiment, cells were cultured at 42 °C for 1 h. In a three-dimensional spheroid assay, tumor spheroids were formed by culturing cells in the U-bottom low-adhesion 96-well plate (S-Bio, Hudson, NH, USA). To evaluate the effect of MSCs or MSC CM, tumor spheroids were grown with MSC spheroids or MSC-derived CM for 48 h.

MSCs were cultured on a collagen-coated culture dish or in suspension with a magnetic stirrer that was rotated at 100 rpm. CM was prepared from 2×10^6^ cells in 9 mL culture medium with antibiotics and a fraction of FBS consisting of 3 kDa or smaller proteins. After one day of incubation, the medium was condensed 10-fold using a filter to collect 3 kDa or heavier proteins (Thermo-Fisher, Waltham, MA, USA).

### *In vitro* assays

Cellular viability was examined using an MTT assay (Invitrogen, Carlsbad, CA, USA) with the procedure previously described 39, as well as an EdU assay with a fluorescence-based cell proliferation kit (Thermo-Fisher, Waltham, MA, USA) 43. The recombinant proteins we employed included Filamin A, Pkm, Pdia3, Tpm4, Anxa2, Eef1a1, Ctsl, Nme2, Dcn, Calr, Aldoa, Calm1, Tpm3, Ppib, Myh9, Ywhae, Hspa5, Hsp90aa1 (MBS962910, MBS8249600, MBS2010131, MBS145304, MBS2009095, MBS2033168, MBS143355, MBS145412, MBS2557309, MBS2009125, MBS8248528, MBS2018713, MBS144696, MBS2009092, MBS717396, MBS143242, MBS806904, MBS142709; MyBioSource, San Diego, CA, USA), Actin Gamma 1, Actn4, Hspa8, Vimentin (H00000071-P01, H00000081-P01, NBP1-30278, NBP2-35139; Novus, Littleton, CO, USA), and Hsp90ab1 (OPCA05157; Aviva system biology, San Diego, CA, USA). A transwell chamber assay was conducted to detect invasive cellular motility 44, and a wound-healing scratch assay was utilized to evaluate 2-dimensional migratory behavior 45. The overexpression of Akt, Lrp5, β-catenin, Snail, Calr, and Ppib was conducted by transfecting plasmids (#10841, #115907, #31785, #31697, # 51161, # 36123; Addgene, Cambridge, MA, USA). RNA interference was conducted using siRNA specific to Akt, Lrp5, β-catenin, Snail, and Hsp90ab1 (65496, s69315, s63417, 69332, s67897, Thermo-Fisher) with a negative siRNA (Silencer Select #1, Thermo-Fisher) as a nonspecific control using the procedure previously described 43.

### Western blot analysis and protein array analysis

Western blot analysis was conducted using the procedure previously described 46. We used antibodies against Lrp5, Runx2, Snail, sclerostin, Calr, p-eIF2α, eIF2α, TGFβ, PDL1 (Cell Signaling, Danvers, MA, USA), LIF, Trail (Novus Biologicals, Centennial, CO, USA), MMP9, NFATc1, cathepsin K (Santa Cruz Biotechnology, Dallas, TX, USA), p53, CXCL2, Ppib (Invitrogen, Carlsbad, CA, USA), Hsp90ab1 (Abcam, Cambridge, UK), and β-actin (Sigma, Saint Louis, MO, USA). We also employed a proteome profiler mouse XL cytokine array kit (R&D Systems, Minneapolis, MN, USA) and determined the expression of 111 cytokines and chemokines in MSC-derived CM.

### *Ex vivo* breast cancer tissue assay

The usage of human breast cancer tissues was approved by the Indiana University Institutional Review Board. A sample (~1 g; ER/PR+, HER2+), received from Simon Cancer Center Tissue Procurement Core, was manually fragmented with a scalpel into small pieces (0.5 ~ 0.8 mm in length). These pieces were grown in DMEM with 10% fetal bovine serum and antibiotics for a day. MSC-derived CM was then added for two additional days, and the change in the fragment size was determined.

### Animal models

The animal procedures were approved by the Indiana University Animal Care and Use Committee and complied with the Guiding Principles in the Care and Use of Animals endorsed by the American Physiological Society. Mice were randomly housed five per cage and provided with mouse chow and water *ad libitum*. In the mouse model of mammary tumors, 8-week old C57BL/6 female mice and BALB/c female mice (10 mice per group; Envigo RMS, Inc.) received subcutaneous injections of EO771 cells and 4T1.2 cells (3.0 × 10^5^ cells in 50 µL PBS), respectively, to the mammary fat pad on day 1 47. In the mouse model of tibial osteolysis, C57BL/6 female mice and BALB/c female mice (10 mice per group) received an injection of EO771 cells and 4T1.2 cells (3.0 × 10^5^ cells in 20 µL PBS), respectively, to the right tibia as an intra-tibial injection.

For examining the anti-tumor efficacy of MSCs, primary mouse MSCs (1.5 × 10^5^ cells in 50 µL PBS), transfected with or without Lrp5 plasmids, were co-injected (3.0 × 10^5^ cells) with EO771 cells to the mammary fat pad. For examining the efficacy of MSC-derived CM, CM was condensed by a filter with a cutoff molecular weight of 3 kDa and the 10-fold condensed CM (50 µL re-suspended in PBS) was intravenously injected from the tail vein. The animals were sacrificed on day 14 and mammary tumors and tibiae were harvested.

### µCT imaging and histology

The tibiae were blindly labeled and analyzed using µCT imaging and histology. Micro-computed tomography was performed using Skyscan 1172 (Bruker-MicroCT, Kontich, Belgium). Using manufacturer-provided software, scans were performed at pixel size 8.99 μm and the images were reconstructed (nRecon v1.6.9.18) and analyzed (CTan v1.13). Using µCT images, trabecular bone parameters such as bone volume ratio (BV/TV), bone mineral density (BMD), trabecular number (Tb.N), and trabecular separation (Tb.Sp.) were determined. In histology, H&E staining was conducted as described previously 44. Of note, normal bone cells appeared in a regular shape with round and deeply stained nuclei, while tumor cells were in a distorted shape with irregularly stained nuclei. X-ray imaging was also conducted with a Faxitron radiographic system (Faxitron X-ray Co.) 48.

### Mass spectrometry-based proteomics analysis

Proteins in CM were analyzed in the Dionex UltiMate 3000 RSLC nano system combined with the Q-exactive high-field hybrid quadrupole orbitrap mass spectrometer (Thermo Fisher Scientific). Proteins were first digested on-beads using trypsin/LysC as described previously 49, 50 except digestion was performed in 50 mM ammonium bicarbonate buffer instead of urea. Digested peptides were then desalted using mini spin C18 spin columns (The Nest Group, Southborough, MA, USA) and separated using a trap and 50-cm analytical columns 49, 51. Raw data were processed using MaxQuant (v1.6.3.3) against the Uniprot mouse protein database at a 1% false discovery rate allowing up to 2 missed cleavages 52. MS/MS counts were used for relative protein quantitation. Proteins identified with at least 1 unique peptide and 2 MS/MS counts were considered for the final analysis.

### Statistical analysis

The number of animals per group was determined based on power analysis to achieve a power of 80% with p < 0.05. For cell-based experiments, three or four independent experiments were conducted and data were expressed as mean ± S.D. Statistical significance was evaluated using a one-way analysis of variance (ANOVA). Post hoc statistical comparisons with control groups were performed using Bonferroni correction with statistical significance at *p* < 0.05. The single and double asterisks in the figures indicate *p* < 0.05 and *p* < 0.01, respectively.

## Supplementary Material

Supplementary methods, figures and table.

## Abbreviations

AKT: Akt kinase (aka protein kinase B)
BMD: bone mineral density
BV/TV: bone volume ratio
Calr: calreticulin
CM: conditioned medium
CXCL2: C-X-C motif ligand 2
DKK1: Dickkopf-related protein 1
DLK1: delta like non-canonical Notch ligand 1
DMEM: Dulbecco's modified Eagle's medium
EdU: 5-ethynyl-2'-deoxyuridine
eIF2α: eukaryotic translation initiation factor 2 alpha
EMT: epithelial-mesenchymal transition
ER: endoplasmic reticulum
GM-CSF: granulocyte-macrophage colony-stimulating factor
H&E: hematoxylin and eosin
Hspa8: heat shock protein family A, member 8
Hsp90aa1: heat shock protein 90 alpha class A, member 1
Hsp90ab1: heat shock protein 90 alpha class B, member 1
IL27: interleukin 27
iTS cells: induced tumor-suppressing cells
LIF: leukemia inhibitory factor
Lrp5: low-density lipoprotein receptor-related protein 5
MMP9: matrix metalloproteinase 9
MRP: mitogen-regulated protein
MSCs: mesenchymal stem cells
PDL-1: programmed death-ligand 1
Ppib: peptidylprolyl isomerase B
Runx2: runt-related transcription factor 2
Tb.N: trabecular number
Tb.Sp: trabecular separation
TGFβ: transforming growth factor beta
