# Preventing tumor progression to the bone by induced tumor-suppressing MSCs: Erratum

**DOI:** 10.7150/thno.77186

**Published:** 2022-08-18

**Authors:** Xun Sun, Kexin Li, Rongrong Zha, Shengzhi Liu, Yao Fan, Di Wu, Misato Hase, Uma K. Aryal, Chien-Chi Lin, Bai-Yan Li, Hiroki Yokota

**Affiliations:** 1 Department of Pharmacology, College of Pharmacy, Harbin Medical University, Harbin 150081, China.; 2 Department of Biomedical Engineering, Indiana University Purdue University Indianapolis, Indianapolis, IN 46202, USA.; 3 Graduate School of Engineering, Mie University, Mie 514, Japan.; 4 Department of Comparative Pathobiology, Purdue University, West Lafayette, IN 47907, USA.; 5 Simon Cancer Center, Indiana University School of Medicine, Indianapolis, IN 46202, USA.; 6 Indiana Center for Musculoskeletal Health, Indiana University School of Medicine, Indianapolis, IN 46202, USA.

The authors regret that the original version of our paper, unfortunately, contained an incorrect picture in Figure 8B, where the images for the Calr case were mistakenly used for the Ppib case. The correct version of Figure 8B is shown below.

The correction made in this erratum does not affect the original data and conclusions. The authors apologize for any inconvenience that the errors may have caused.

## Figures and Tables

**Figure 8:**
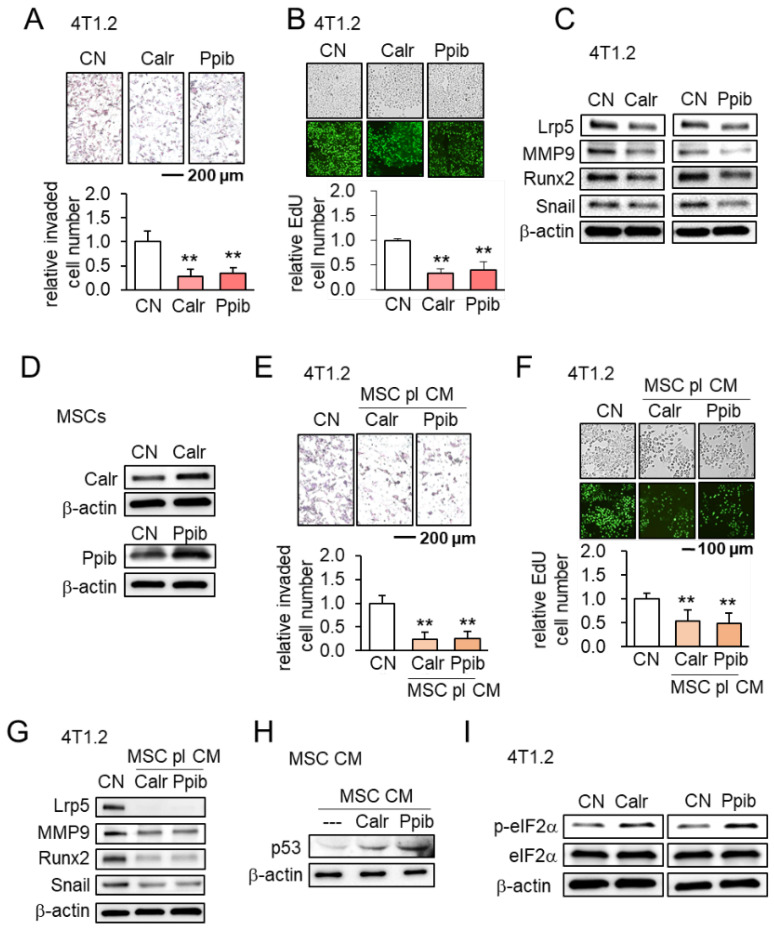
Effects of Calr and Ppib on 4T1.2 mammary tumor cells. The double asterisk indicates p < 0.01. CN = control, and CM = conditioned medium. **(A-B)** Reduction in transwell invasion and EdU-based proliferation by recombinant Calr and Ppib proteins. **(C)** Reduction in Lrp5, MMP9, Runx2 and Snail by recombinant Calr and Ppib proteins. **(D)** Overexpression of Calr and Ppib in MSCs. **(E-F)** Reduction in Transwell invasion and EdU-based proliferation of 4T1.2 cells in response to Calr- and Ppib-overexpressing MSC CM. **(G)** Downregulation of Lrp5, MMP9, Runx2 and Snail in 4T1.2 cells in response to Calr- and Ppib-overexpressing MSC CM. **(H)** Upregulation of p53 in Calr- and Ppib-overexpressing MSC CM. **(I)** Elevation of phosphorylated eIF2a in 4T1.2 cells in response to Calr and Ppib recombinant proteins.

