# Supplementary material for: Preventing tumor progression to the bone by induced tumor-suppressing MSCs
Source: Theranostics. 2021 Mar 5;11(11):5143–59. doi: 10.7150/thno.58779 (PMC8039940; doi:10.7150/thno.58779)
Supplement: Supplementary file 1 — Supplementary methods, figures and table. [file thnov11p5143s1.pdf]

Supplementary Methods

Cell culture. EO771 mouse mammary tumor cells (CH3 BioSystems, Amherst, NY, USA) [1], 4T1.2 mouse mammary tumor cells (obtained from Dr. R. Anderson at Peter MacCallum Cancer Institute, Australia) [2], and MDA-MB-231 breast cancer cells (ATCC) were cultured in DMEM. TRAMP-C2ras prostate tumor cells (ATCC) were cultured in DMEM/F-12 [3]. Murine MSCs derived from the bone marrow of the C57BL/6 strain (Envigo RMS, Inc., Indianapolis, IN, USA) were cultured in MesenCult culture medium (Stem Cell Technology, Cambridge, MA, USA). The culture media was supplemented with 10% fetal bovine serum and antibiotics, and cells were maintained at 37°C and 5% CO_2. In a heat shock experiment, cells were cultured at 42 °C for 1 h. In a three-dimensional spheroid assay, tumor spheroids were formed by culturing cells in the U-bottom low-adhesion 96-well plate (S-Bio, Hudson, NH, USA). To evaluate the effect of MSCs or MSC CM, tumor spheroids were grown with MSC spheroids or MSC-derived CM for 48 h.

MSCs were cultured on a collagen-coated culture dish or in suspension with a magnetic stirrer that was rotated at 100 rpm. CM was prepared from 2×10^6 cells in 9 mL culture medium with antibiotics and a fraction of FBS consisting of 3 kDa or smaller proteins. After one day of incubation, the medium was condensed 10-fold using a filter to collect 3 kDa or heavier proteins (Thermo-Fisher, Waltham, MA, USA).

***In vitro* assays**. Cellular viability was examined using an MTT assay (Invitrogen, Carlsbad, CA, USA) with the procedure previously described [4], as well as an EdU assay with a fluorescence-based cell proliferation kit (Thermo-Fisher, Waltham, MA, USA) [5]. The recombinant proteins

we employed included HSF1, Hsp90aa1 (MBS2018330, MBS142709; MyBioSource, San Diego, CA, USA). A transwell chamber assay was conducted to detect invasive cellular motility [6], and a wound-healing scratch assay was utilized to evaluate 2-dimensional migratory behavior [7]. The overexpression of Akt, Lrp5, β-catenin and Snail was conducted by transfecting plasmids (#10841, #115907, #31785, #31697; Addgene, Cambridge, MA, USA). RNA interference was conducted using siRNA specific to Akt, Lrp5, β-catenin and Snail (65496, s69315, s63417, 69332, Thermo-Fisher) with a negative siRNA (Silencer Select #1, Thermo-Fisher) as a nonspecific control using the procedure previously described [5].

Western blot analysis and protein array analysis. Western blot analysis was conducted using the procedure previously described [8]. We used antibodies against Lrp5, Runx2, Snail, Calr, p-Akt and Akt (Cell Signaling, Danvers, MA, USA), LIF, Trail (Novus Biologicals, Centennial, CO, USA), MMP9 (Santa Cruz Biotechnology, Dallas, TX, USA), p53, CXCL2, Ppib (Invitrogen, Carlsbad, CA, USA), Hsp90ab1 (Abcam, Cambridge, UK), and β-actin (Sigma, Saint Louis, MO, USA).

Mass spectrometry-based proteomics analysis. Proteins in CM were analyzed in the Dionex UltiMate 3000 RSLC nano system combined with the Q-exactive high-field hybrid quadrupole orbitrap mass spectrometer (Thermo Fisher Scientific). Proteins were first digested on-beads using trypsin/LysC as described previously [9, 10] except digestion was performed in 50 mM ammonium bicarbonate buffer instead of urea. Digested peptides were then desalted using mini spin C18 spin columns (The Nest Group, Southborough, MA, USA) and separated using a trap and 50-cm analytical columns [9, 11]. Raw data were processed using MaxQuant (v1.6.3.3)

against the Uniprot mouse protein database at a 1% false discovery rate allowing up to 2 missed cleavages [12]. MS/MS counts were used for relative protein quantitation. Proteins identified with at least 1 unique peptide and 2 MS/MS counts were considered for the final analysis.

Statistical analysis. The number of animals per group was determined based on power analysis to achieve a power of 80% with $p < 0.05$. For cell-based experiments, three or four independent experiments were conducted and data were expressed as mean ± S.D. Statistical significance was evaluated using a one-way analysis of variance (ANOVA). Post hoc statistical comparisons with control groups were performed using Bonferroni correction with statistical significance at $p < 0.05$. The single and double asterisks in the figures indicate $p < 0.05$ and $p < 0.01$, respectively.

Supplementary References

1. Ewens A, Mihich E, Ehrke MJ. Distant metastasis from subcutaneously grown E0771 medullary breast adenocarcinoma. Anticancer research. 2005; 25: 3905-15.
2. Lelekakis M, Moseley JM, Martin TJ, Hards D, Williams E, Ho P, et al. A novel orthotopic model of breast cancer metastasis to bone. Clinical & experimental metastasis. 1999; 17: 163-70.
3. Mikyskova R, Stepanek I, Indrova M, Bieblova J, Simova J, Truxova I, et al. Dendritic cells pulsed with tumor cells killed by high hydrostatic pressure induce strong immune responses and display therapeutic effects both in murine TC-1 and TRAMP-C2 tumors when combined with docetaxel chemotherapy. International journal of oncology. 2016; 48: 953-64.
4. Chen A, Wang L, Li BY, Sherman J, Ryu JE, Hamamura K, et al. Reduction in Migratory Phenotype in a Metastasized Breast Cancer Cell Line via Downregulation of S100A4 and GRM3. Scientific reports. 2017; 7: 3459.
5. Liu S, Fan Y, Chen A, Jalali A, Minami K, Ogawa K, et al. Osteocyte-Driven Downregulation of Snail Restrains Effects of Drd2 Inhibitors on Mammary Tumor Cells. Cancer research. 2018; 78: 3865-76.
6. Xu W, Wan Q, Na S, Yokota H, Yan JL, Hamamura K. Suppressed invasive and migratory behaviors of SW1353 chondrosarcoma cells through the regulation of Src, Rac1 GTPase, and MMP13. Cellular signalling. 2015; 27: 2332-42.
7. Liu S, Liu Y, Minami K, Chen A, Wan Q, Yin Y, et al. Inhibiting checkpoint kinase 1 protects bone from bone resorption by mammary tumor in a mouse model. Oncotarget. 2018; 9: 9364-78.
8. Li F, Chen A, Reeser A, Wang Y, Fan Y, Liu S, et al. Vinculin Force Sensor Detects Tumor-Osteocyte Interactions. Scientific reports. 2019; 9: 5615.
9. Connelly KE, Hedrick V, Paschoal Sobreira TJ, Dykhuizen EC, Aryal UK. Analysis of Human Nuclear Protein Complexes by Quantitative Mass Spectrometry Profiling. Proteomics. 2018; 18: e1700427.
10. Opoku-Temeng C, Onyedibe KI, Aryal UK, Sintim HO. Proteomic analysis of bacterial response to a 4-hydroxybenzylidene indolinone compound, which re-sensitizes bacteria to traditional antibiotics. Journal of proteomics. 2019; 202: 103368.
11. Howe EN, Burnette MD, Justice ME, Schnepp PM, Hedrick V, Clancy JW, et al. Rab11b-mediated integrin recycling promotes brain metastatic adaptation and outgrowth. Nature communications. 2020; 11: 3017.
12. Cox J, Mann M. MaxQuant enables high peptide identification rates, individualized p.p.b.-range mass accuracies and proteome-wide protein quantification. Nature biotechnology. 2008; 26: 1367-72.

Supplementary Figures and Supplementary Tables

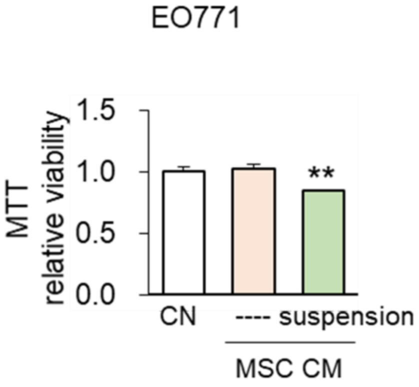

Figure S1. Effects of MSC CM in suspension culture and RNA interference with vinculin siRNA on the MTT-based viability of EO771 mammary tumor cells. The double asterisk indicates p < 0.01.

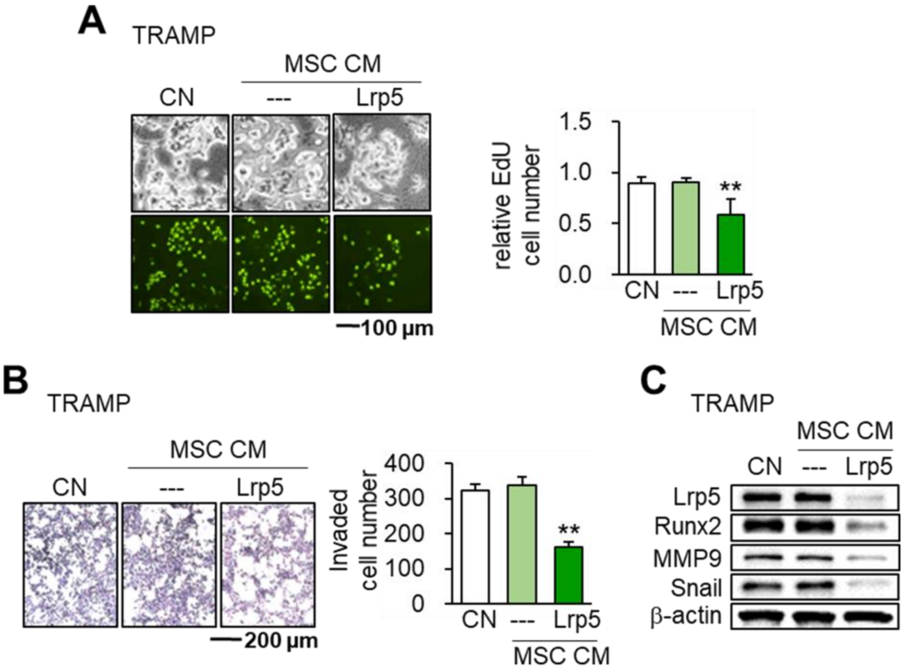

Figure S2. Effects of Lrp5-overexpressing MSC CM on the behaviors of TRAMP prostate tumor cells. The double asterisk indicates p < 0.01. **(A-B)** Reduction in EdU-based proliferation and transwell-based invasion of TRAMP cells in response to Lrp5-overexpressing MSC CM. **(C)** Reduced expression levels of Lrp5, Runx2, MMP9, and Snail in TRAMP cells in response to Lrp5-overexpressing MSC CM.

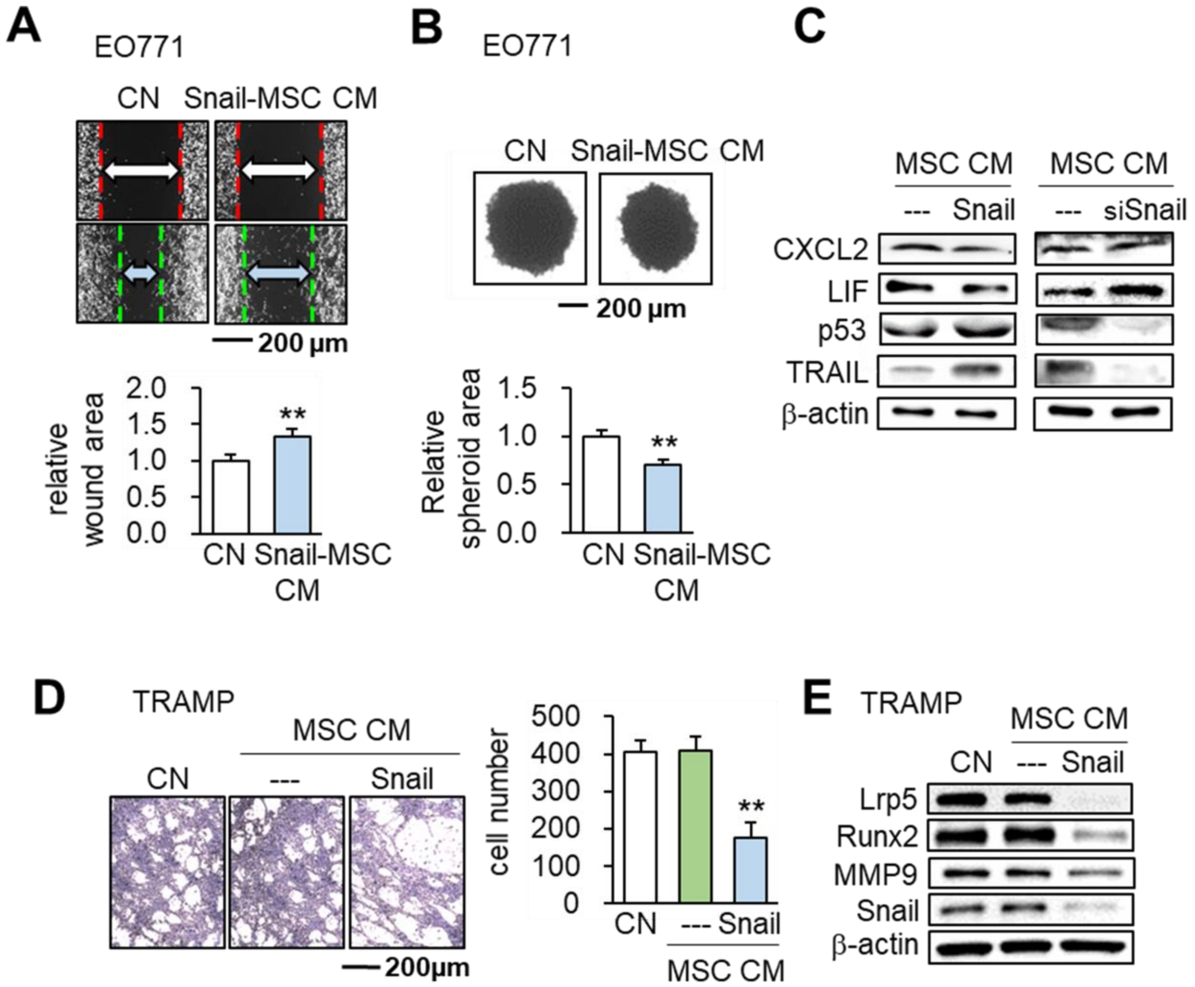

Figure S3. Effect of Snail-overexpressing MSC CM on EO771 mammary tumor cells and TRAMP prostate tumor cells. Snail-MSC CM = Snail-overexpressing MSC CM, and siSnail = Snail siRNA. The double asterisk indicates $p < 0.01$. **(A-B)** Reduction in scratch-based migration and three-dimensional EO771 tumor spheroid size in response to Snail-overexpressing MSC CM. **(C)** Downregulation of CXCL2 and LIF, and upregulation of p53 and Trail by Snail-overexpressing MSC CM. The responses were reversed by Snail-silenced MSC CM. **(D)** Reduction in the Transwell-based invasion of TRAMP cells in response to Snail-overexpressing MSC CM. **(E)** Reduced expression levels of Lrp5, Runx2, MMP9, and Snail in TRAMP prostate tumor cells in response to Snail-overexpressing MSC CM.

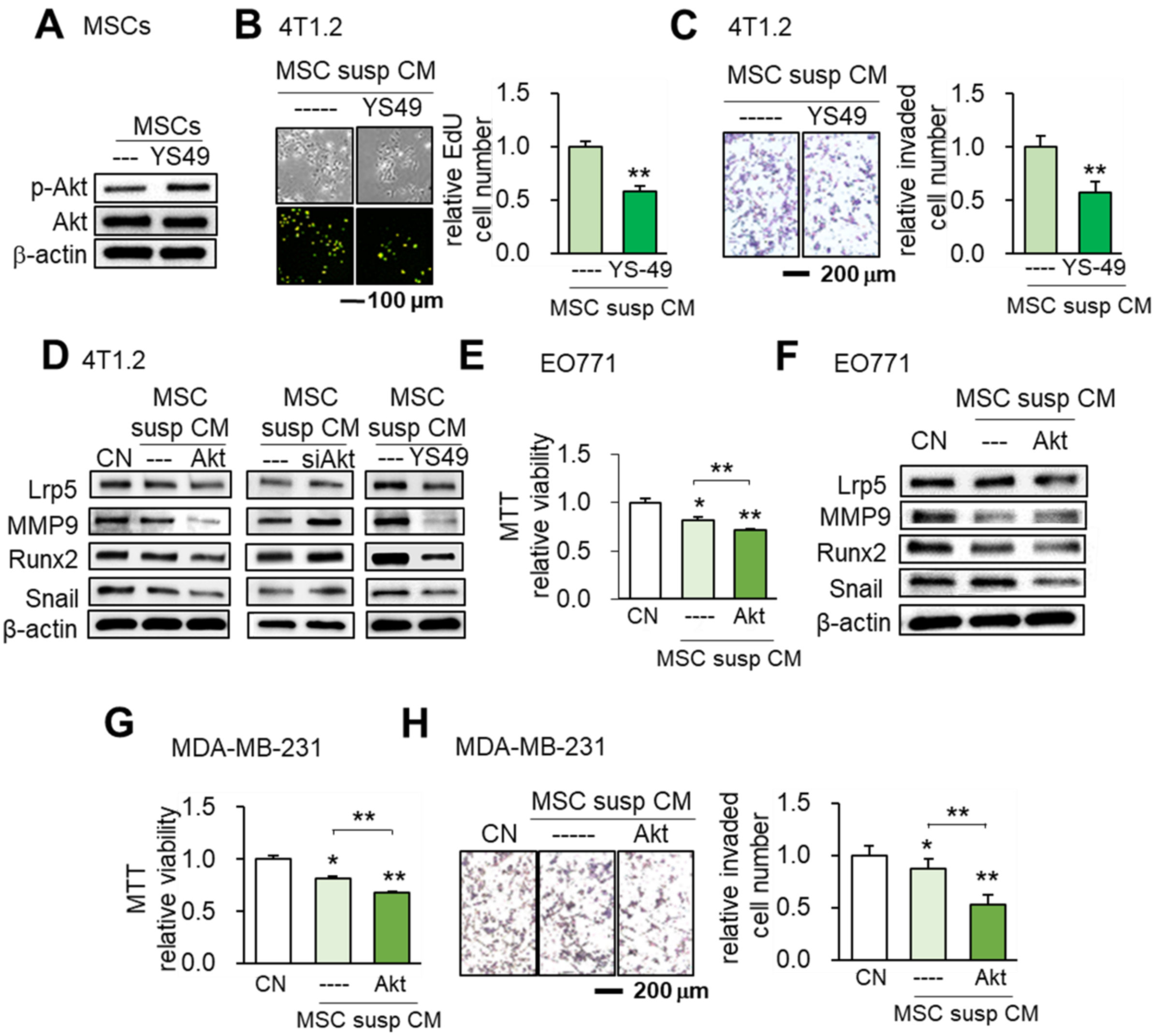

Figure S4. Effects of Akt-overexpressing/silenced MSC CMs and YS49 on 4T1.2, EO771, and MDA-MB-231 cancer cells. Of note, susp = suspension culture, Akt pl = Akt plasmids, siAkt = Akt siRNA. The single and double asterisks indicate $p < 0.05$ and 0.01, respectively. **(A-C)** Elevation of phosphorylated Akt by YS49, and a significant reduction in EdU-based proliferation and transwell-based invasion of 4T1.2 cells by YS49-treated MSC CM. **(D)** Altered expression levels of Lrp5, MMP9, Runx2, TGFβ, and Snail in response to Akt-overexpressing/silenced or YS49-treated MSC CMs. **(E)** Reduction in MTT-based viability of EO771 mammary tumor cells by MSC CM in suspension culture and Akt-overexpressing MSC CM. **(F)** Reduced expression levels of Lrp5, MMP9, Runx2, Snail, and TGFβ in EO771 cells in response to Akt-overexpressing MSC CM. **(G-H)** Reduction in MTT-based viability and transwell-based invasion of MDA-MB-231 breast cancer cells by MSC CM in suspension culture and Akt-overexpressing MSC CM.

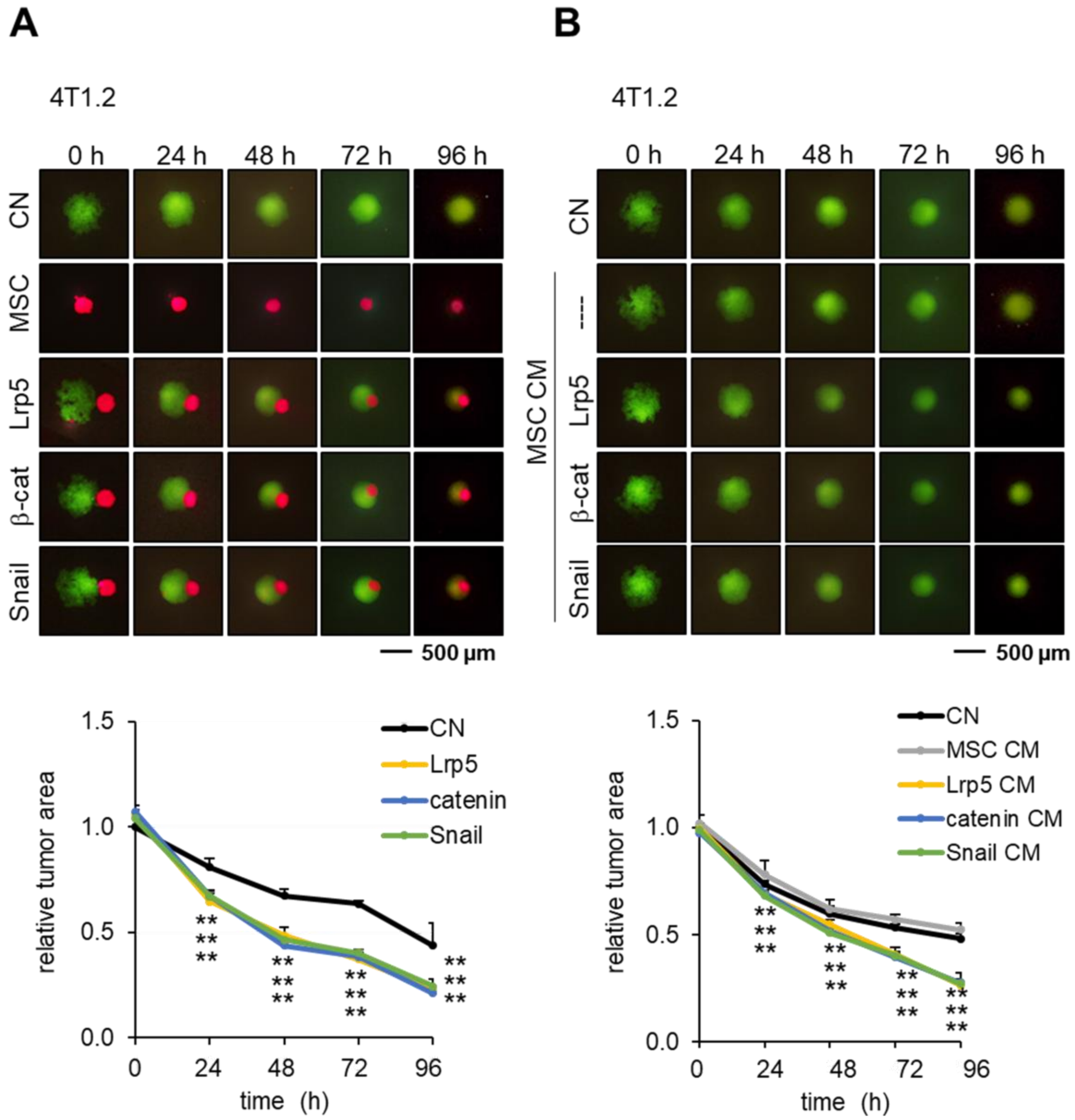

Figure S5. Cell competition assay for 4T1.2 tumor spheroids in response to MSC spheroids and MSC CMs, in which Lrp5, β-catenin or Snail was overexpressed. **(A)** Reduction in three-dimensional 4T1.2 tumor spheroids in response to Lrp5-, β-cat-, and Snail-overexpressing MSCs. **(B)** Reduction in three-dimensional 4T1.2 tumor spheroids in response to Lrp5-, β-catenin, and Snail-overexpressing MSC CMs.

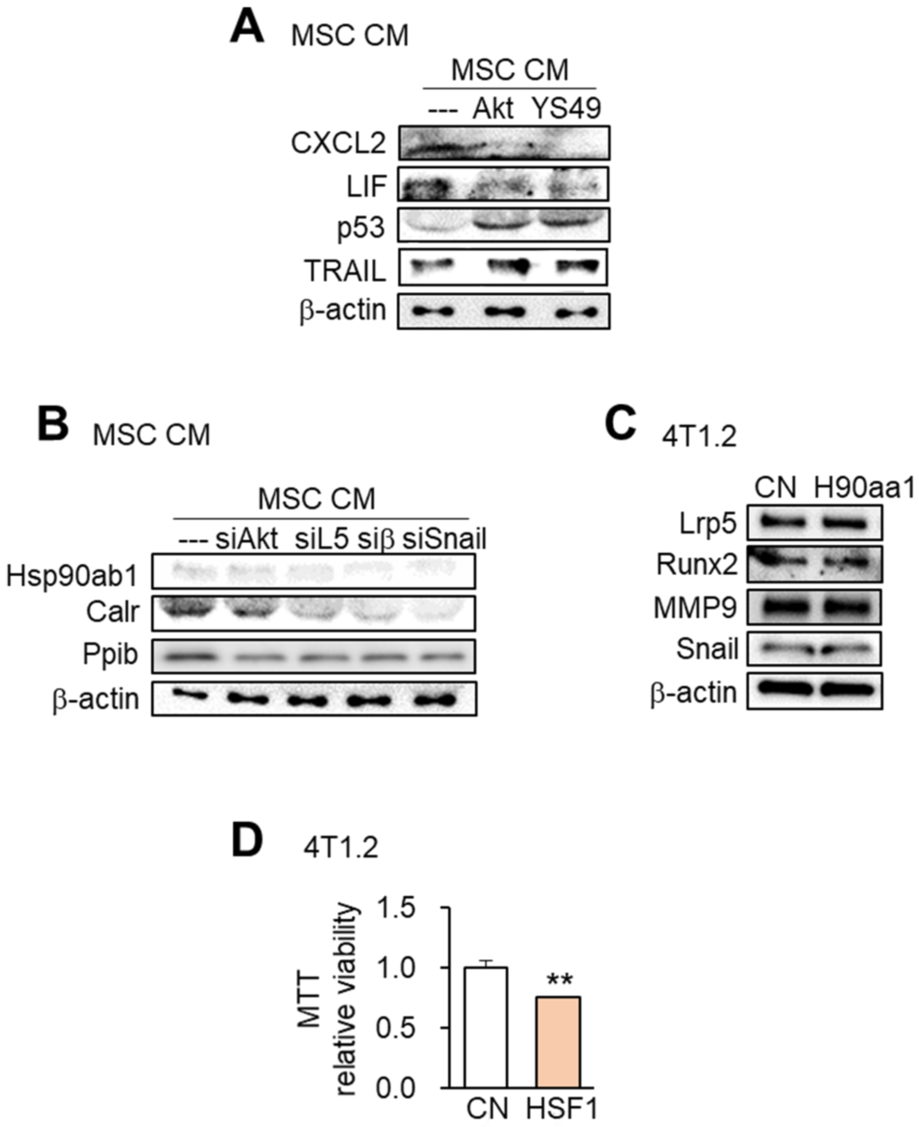

Figure S6. Effects of Akt, YS49, Hsp90ab1, Calr, Ppib, and Hsp90aa1. CN = control, and C = conditioned medium. **(A)** Downregulation of CXCL2 and LIF and upregulation of p53 and Trail in Akt-overexpressing and YS49-treated MSC CMs. **(B)** Reduction of Hsp90ab1, Calr, and Ppib in MSC CM by the silencing of Akt, Lrp5, β-catenin, and Snail. **(C)** Undetectable change of Lrp5, Runx2, MMP9, and Snail in 4T1.2 cells in response to H90aa1 recombinant proteins. **(D)** Reduction in the MTT-based cell viability of 4T1.2 cells in response to HSF1 recombinant proteins.

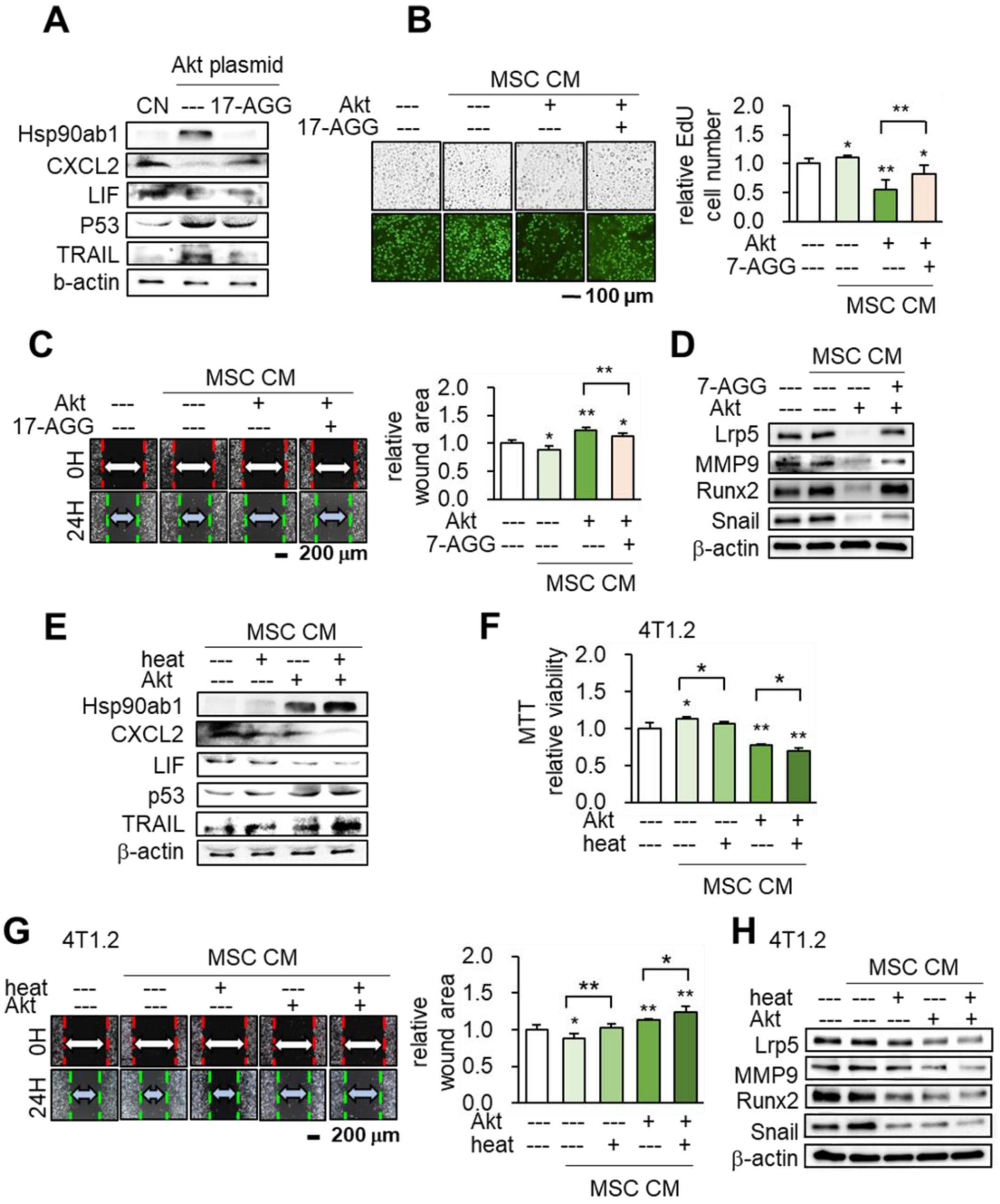

Figure S7. Hsp90ab1 as an extracellular tumor suppressor in 4T1.2 mammary tumor cells. The single and double asterisks indicate $p < 0.05$ and 0.01, respectively. CN = control, CM = conditioned medium, Akt = Akt overexpression, 17-AGG = Hsp90 inhibitor (Tanespimycin), and heat = heat shock at 42°C for 1 h. **(A)** Regulation of Hsp90ab1, CXCL2, LIF, p53, and Trail by the inhibition of Hsp90. **(B-C)** Suppression of MSC CM-driven reduction in the EdU-based proliferation and scratch-based migration by the inhibition of Hsp90. **(D)** Suppression of MSC CM-driven reduction in Lrp5, MMP9, Runx2, and Snail by the inhibition of Hsp90. **(E-H)** Enhancement of the tumor-suppressing action of MSC CM by heat shock at 42°C for 1 h.

Suppl. Table S1

List of 75 proteins that were expressed higher in Akt-overexpressing and YS49-treated CMs than the control CM in mass spectrometry-based proteomics analysis.

Num	Gene names	Mol [kDa]	CN	AKT	YS49	Num	Gene names	Mol [kDa]	CN	AKT	YS49
1	Hspa5	72.4	0	25	45	39	Arhgdia	23.4	0	4	4
2	Actg1	41.8	20	40	221	40	EG433182;Eno1	47.1	8	11	116
3	Flna	280.5	0	18	83	41	Atp5b	56.3	0	3	72
4	Vim	53.7	3	20	51	42	Plec	506.5	0	3	71
5	Hsp90ab1	83.3	15	27	101	43	Eif4a1	46.0	0	3	49
6	Hspa8	70.9	8	19	160	44	Gm1821;Ubc;Gm8797;Uba52;Rps27a;Kxd1;Ubb	17.2	3	6	28
7	Actn4	105.0	4	14	27						
8	Pkm	57.8	8	17	133	45	Lmna	74.2	0	3	22
9	P4hb	56.6	0	9	7	46	Eef1g	50.1	0	3	16
10	Pdia3	56.7	0	9	6	47	Ctsb	37.3	9	12	17
11	Tpm4	28.5	0	9	4	48	Got1	46.2	2	5	10
12	Anxa2	38.6	3	11	72	49	GAPDH;Gapdh;Gm3839	35.8	0	3	8
13	Msn	67.7	0	8	9	50	Actg1	43.6	0	3	4
14	Eef1a1	50.1	4	11	100	51	Eef2	95.3	0	2	48
15	Ctsl	37.6	0	7	14	52	Flnb	277.8	0	2	42
16	Gm20390;Nme2;Nme1	30.2	4	11	9	53	Rps3	26.7	0	2	35
17	Dcn	39.8	0	7	5	54	Pgk1	44.6	2	4	36
18	Calr	42.2	14	20	30	55	Hnrnpa2b1	37.4	0	2	25
19	Aldoa	39.4	0	6	20	56	Hsp90b1	92.5	0	2	13
20	Calm1	16.8	3	9	20	57	Mif	12.5	2	4	14
21	Tpm3;Tpm3-rs7	29.0	2	8	12	58	Pgam1	28.8	2	4	11
22	Ppib	23.7	0	6	9	59	Ppia	18.0	0	2	8
23	Tuba1b;Tuba1c;Tuba1a	50.2	2	7	166	60	Hmgb1	19.8	0	2	6
24	Myh9	226.4	0	5	87	61	Prdx2	21.8	0	2	6
25	Ywhae	29.2	14	19	38	62	Pdia4	72.4	0	2	6
26	Hsp90aa1	84.8	0	5	32	63	Psmb1	15.6	0	2	5
27	Vcp	89.3	7	12	27	64	Sh3d21	26.8	16	18	20
28	Actn1	103.1	0	5	13	65	Ybx1	35.7	0	2	4
29	Psma7;Psma8	27.9	0	5	13	66	Actb	41.8	0	2	4
30	Actb;Actg1	41.8	0	5	2	67	Pdia6	48.7	0	2	4
31	Tubb5	49.7	3	7	116	68	Akr1a1	36.6	0	2	4
32	Got2	47.4	6	10	20	69	Marcks	29.7	2	4	5
33	Mdh2	35.6	0	4	13	70	Psma1	29.5	0	2	3
34	Calu	37.1	0	4	13	71	Tmsb4x	5.1	0	2	3
35	Prdx1	22.2	0	4	11	72	Ahnak	604.3	0	2	2
36	Ncl	76.9	2	6	11	73	Ywhag	28.3	2	4	3
37	Hspa4	94.2	0	4	9	74	Set;BC085271	17.6	2	3	6
38	C3	186.5	11	15	18	75	Aga	37.0	2	3	6
